# Supplementary material for: Insertion of an Amphipathic Linker in a Tetrapodal Tryptophan Derivative Leads to a Novel and Highly Potent Entry Inhibitor of Enterovirus A71 Clinical Isolates
Source: Int J Mol Sci. 2023 Feb 10;24(4):3539. doi: 10.3390/ijms24043539 (PMC9959982; doi:10.3390/ijms24043539)
Supplement: Supplementary file 1 [file ijms-24-03539-s001.zip › ijms-2132794-supplementary.pdf]

Supplementary material for publication online

INSERTION OF AN AMPHIPATHIC LINKER IN A TETRAPODAL TRYPTOPHAN DERIVATIVE LEADS TO A NOVEL AND HIGHLY POTENT ENTRY INHIBITOR OF ENTEROVIRUS A71 CLINICAL ISOLATES

Olaia Martí-Marí [1], Rana Abdelnabi [2], Dominique Schols [2], Johan Neyts [2], María-José Camarasa [1], Federico Gago [3,*] and Ana San-Félix [1,*]

[1] Instituto de Química Médica (IQM, CSIC), E-28006 Madrid, Spain
[2] Laboratory of Virology and Chemotherapy, Rega Institute for Medical Research, Department of Microbiology and Immunology, University of Leuven, B-3000 Leuven, Belgium
[3] Departamento de Ciencias Biomédicas y Unidad Asociada IQM-UAH, Universidad de Alcalá, E-28805 Alcalá de Henares, Spain
* Correspondence: federico.gago@uah.es (F.G.); anarosa@iqm.csic.es (A.S.-F.); Tel.: +34-918854514 (F.G.); +34-912587617 (A.S.-F.)

Table of Contents

S2–S33: Representative ^{1}H, ^{13}CNMR, MS and HPLC spectra

S34: Supplementary **Figure S1**: (**A**) Top view of the VP1 pentamer (each subunit in a different color) showing the ten top-scoring poses for the decorated Trp (sticks, C atoms in grey) present in **AL-470** and **AL-471** bound at two distinct subunit interfaces. (**B**) Enlarged view of a representative docked fragment of **AL-470** and **AL-471**. (**C**) Binding pose of the D-Trp counterpart of the same fragment.

S35: Supplementary **Figure S2**: Side view of the VP1 pentamer (each subunit in a different color) showing the three top-scoring binding poses for the decorated Trp present in **AL-534** (sticks, C atoms in brown). The corresponding fragment from **AL-471** (sticks, C atoms in grey) is also displayed for comparison.

S36: Supplementary **Figure S3**: (**A**) Root-mean-square deviations from the initial pose for VP1-bound **AL-534** (whole molecule, blue; two protein-bound "pendant legs", green; the "pendant leg" shown in Fig. S2, orange). (**B**) Atomic fluctuations per residue (solid circles, all atoms; open triangles, Cα atoms) in VP1 (each subunit [residues 78-280] in a different color) throughout the MD simulations.

S37: Predicted pharmacokinetic and toxicological properties for **23** (**AL-534**), **AL-471**, and pirodavir (https://biosig.lab.uq.edu.au/pkcsm/prediction).

Representative ^{1}H, ^{13}CNMR, MS and HPLC spectra

Intermediate 2

^{1}H NMR

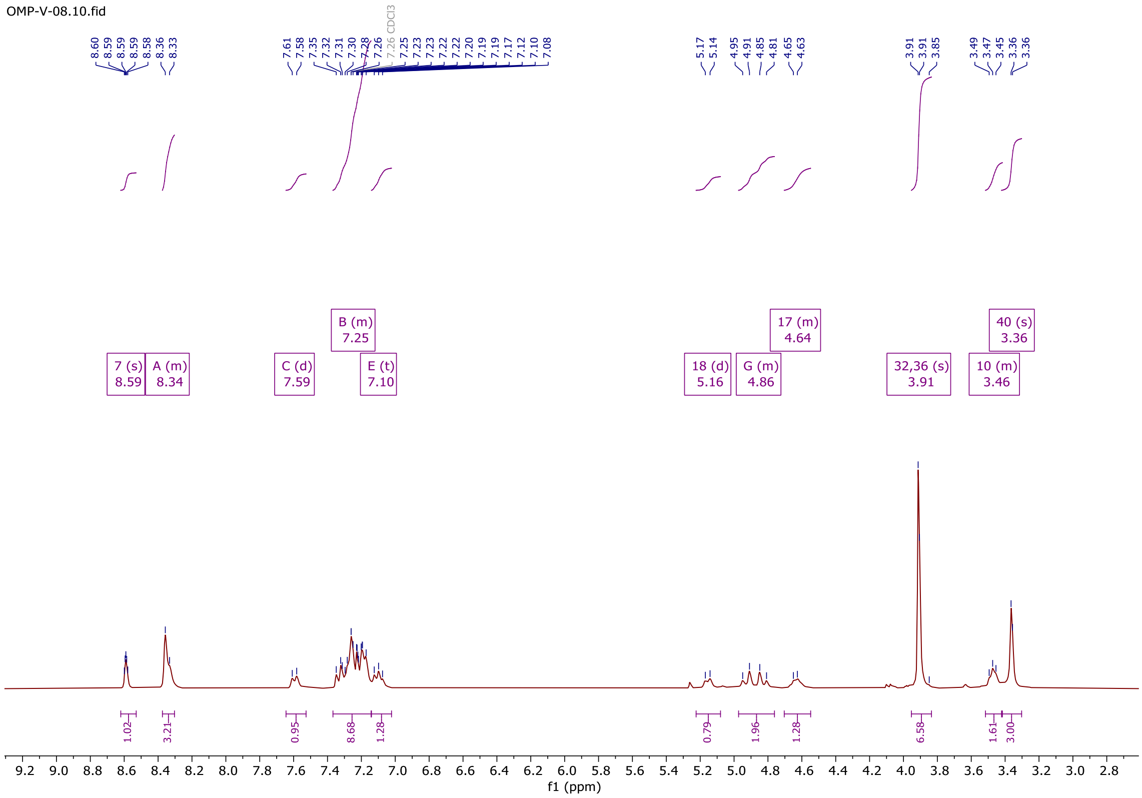
OMP-V-08.10.fid
8.60 8.59 8.59 8.59 8.58 8.36 8.33
7.61 7.58 7.35 7.32 7.31 7.30 7.28 7.26 CDCl3 7.25 7.23 7.23 7.22 7.22 7.20 7.19 7.19 7.17 7.12 7.10 7.08
5.17 5.14 4.95 4.91 4.85 4.81 4.65 4.63
3.91 3.91 3.85
3.49 3.47 3.45 3.36 3.36
7 (s) 8.59
A (m) 8.34
C (d) 7.59
B (m) 7.25
E (t) 7.10
18 (d) 5.16
G (m) 4.86
17 (m) 4.64
32,36 (s) 3.91
10 (m) 3.46
40 (s) 3.36
1.02
3.21
0.95
8.68
1.28
0.79
1.96
1.28
6.58
1.61
3.00
9.2 9.0 8.8 8.6 8.4 8.2 8.0 7.8 7.6 7.4 7.2 7.0 6.8 6.6 6.4 6.2 6.0 5.8 5.6 5.4 5.2 5.0 4.8 4.6 4.4 4.2 4.0 3.8 3.6 3.4 3.2 3.0 2.8
f1 (ppm)

Tetramer 5

^{1}H NMR

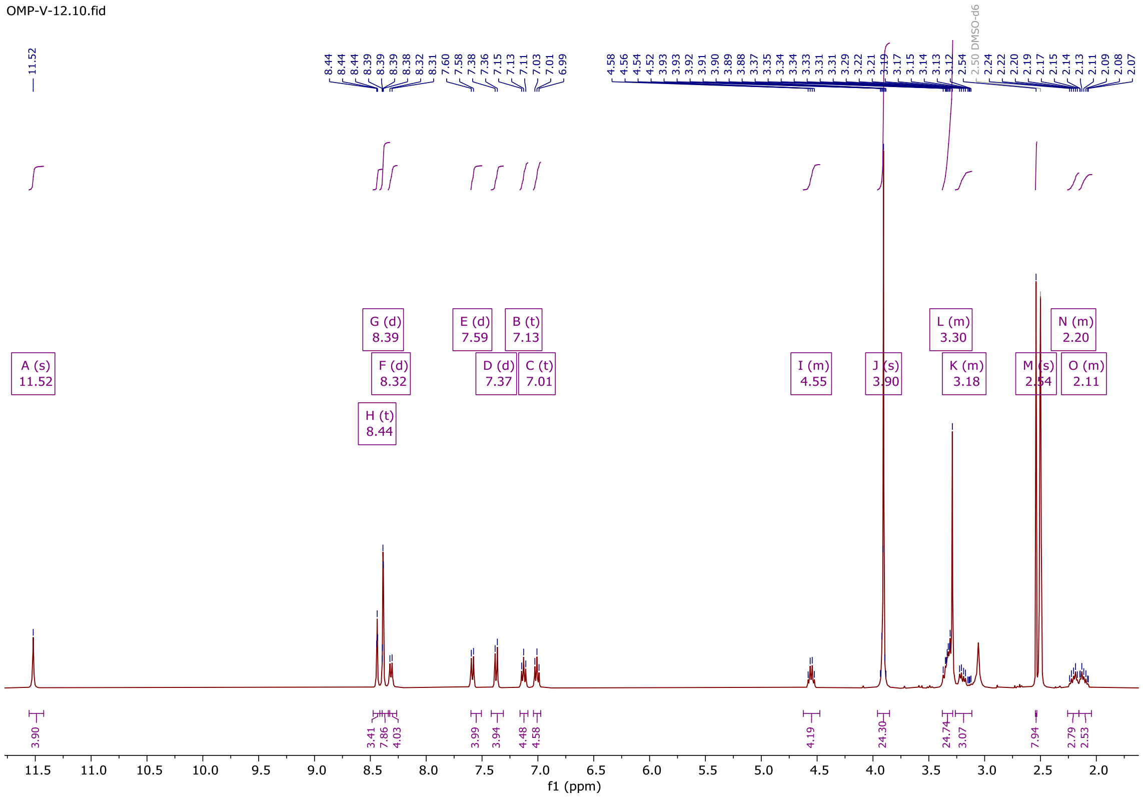

Tetramer 6

^{1}H NMR

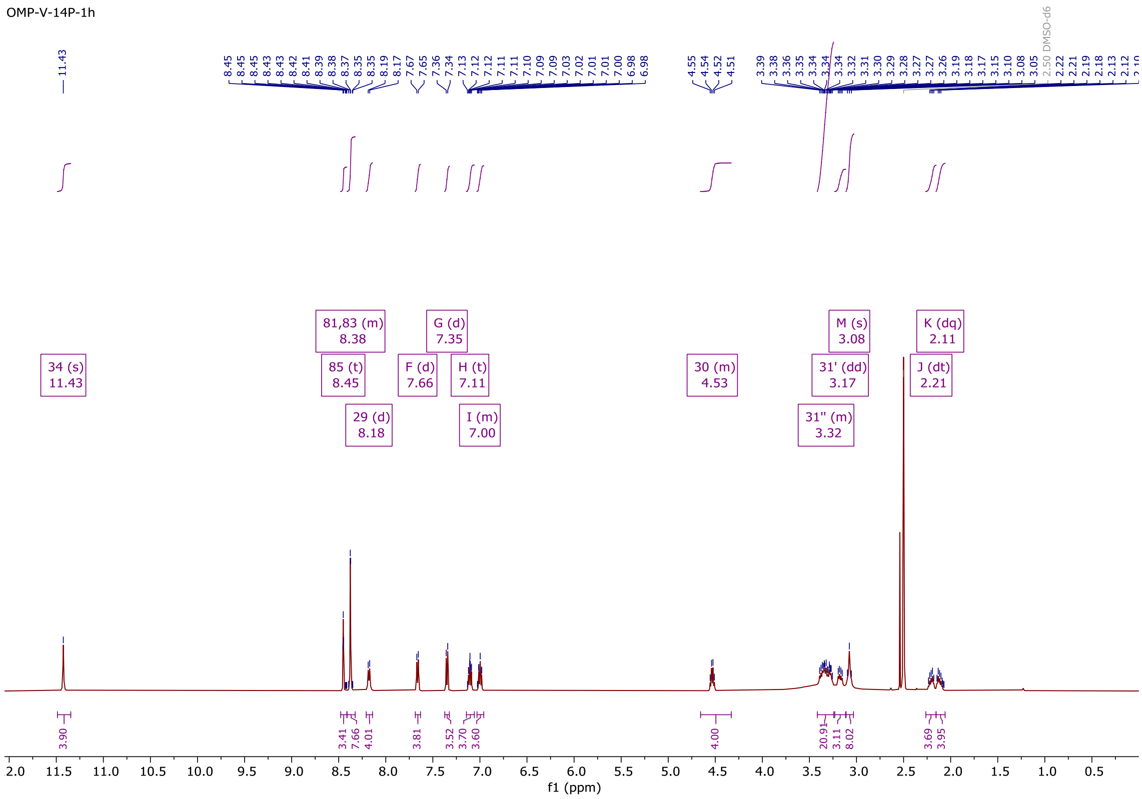

^{13}C NMR

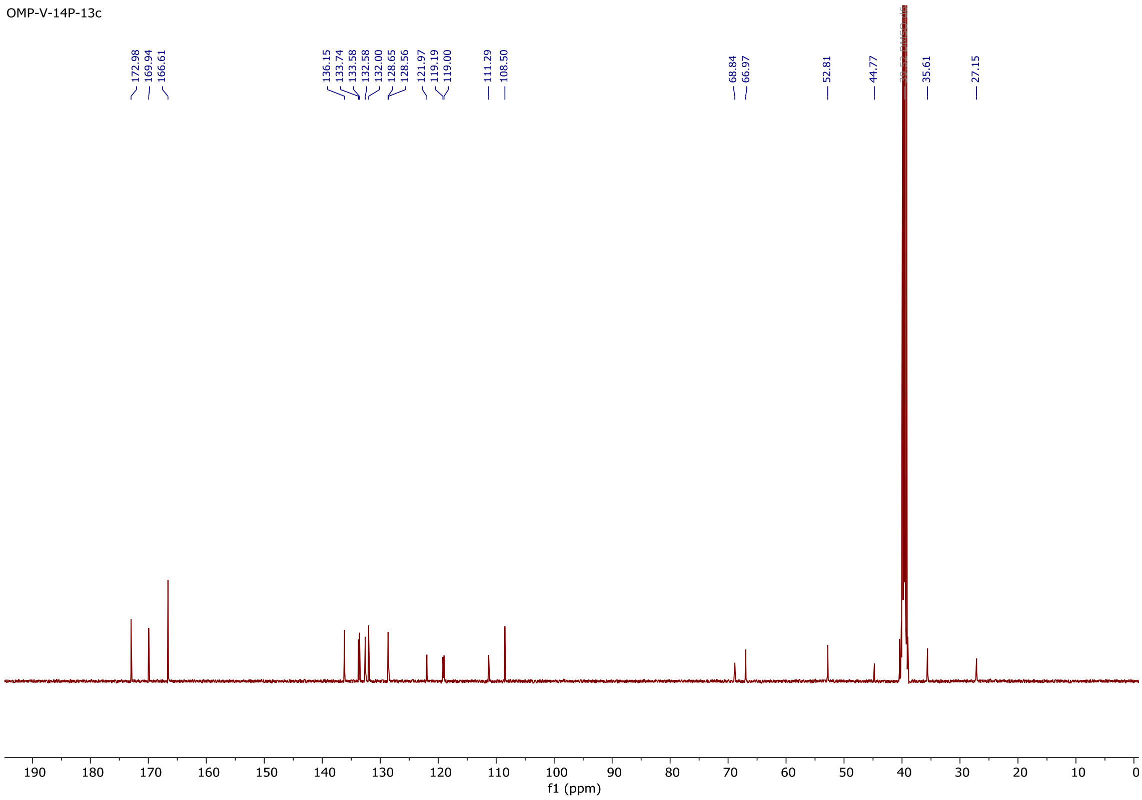

Intermediate 8

^{1}H NMR

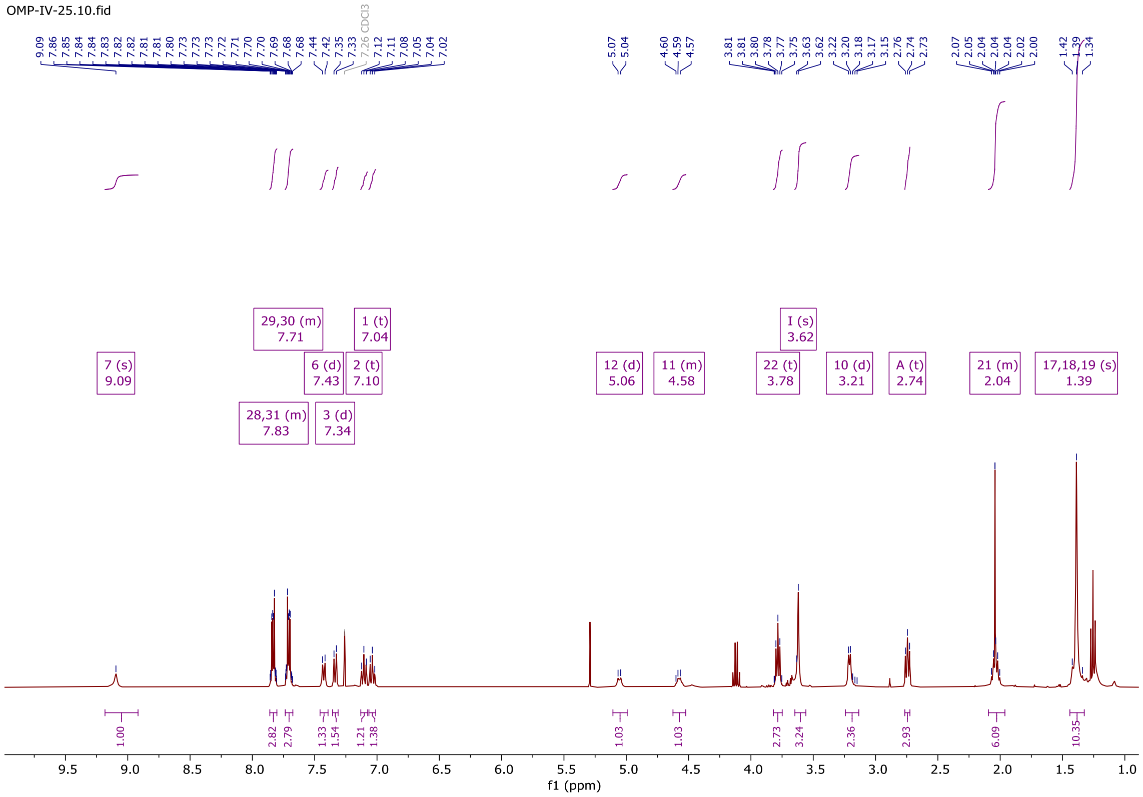

^{13}C NMR

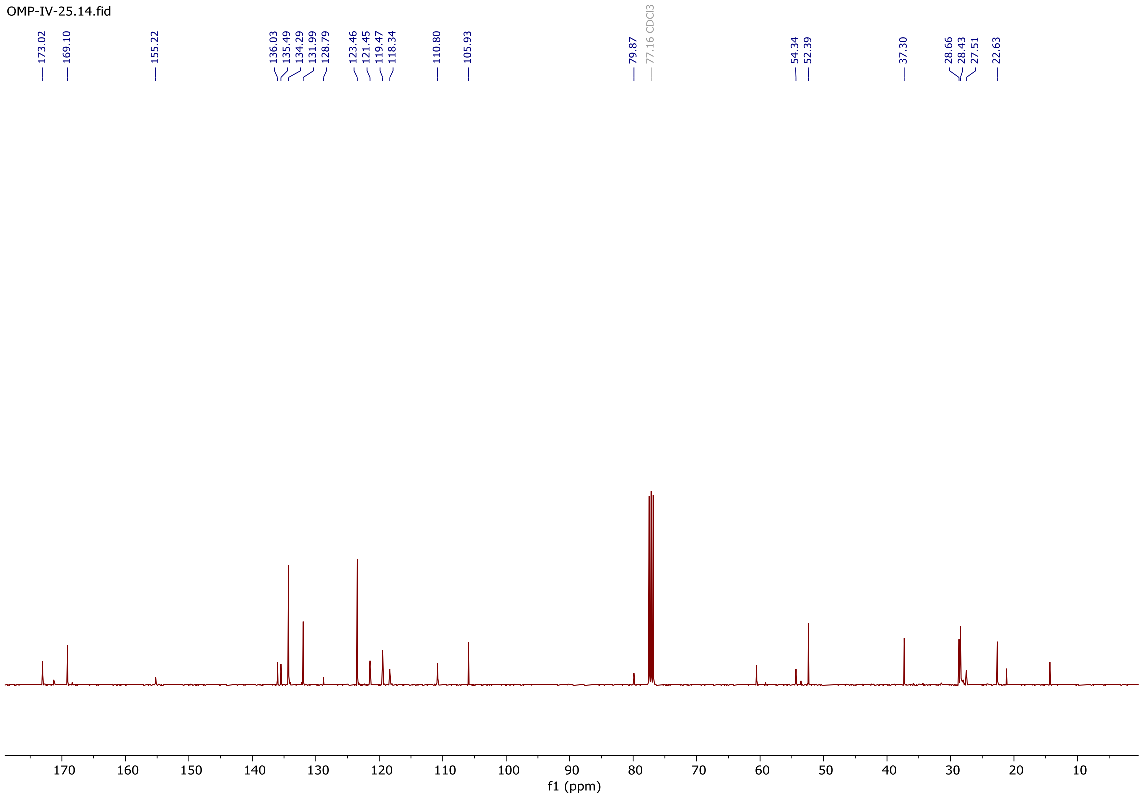

Intermediate 9

^{1}H NMR

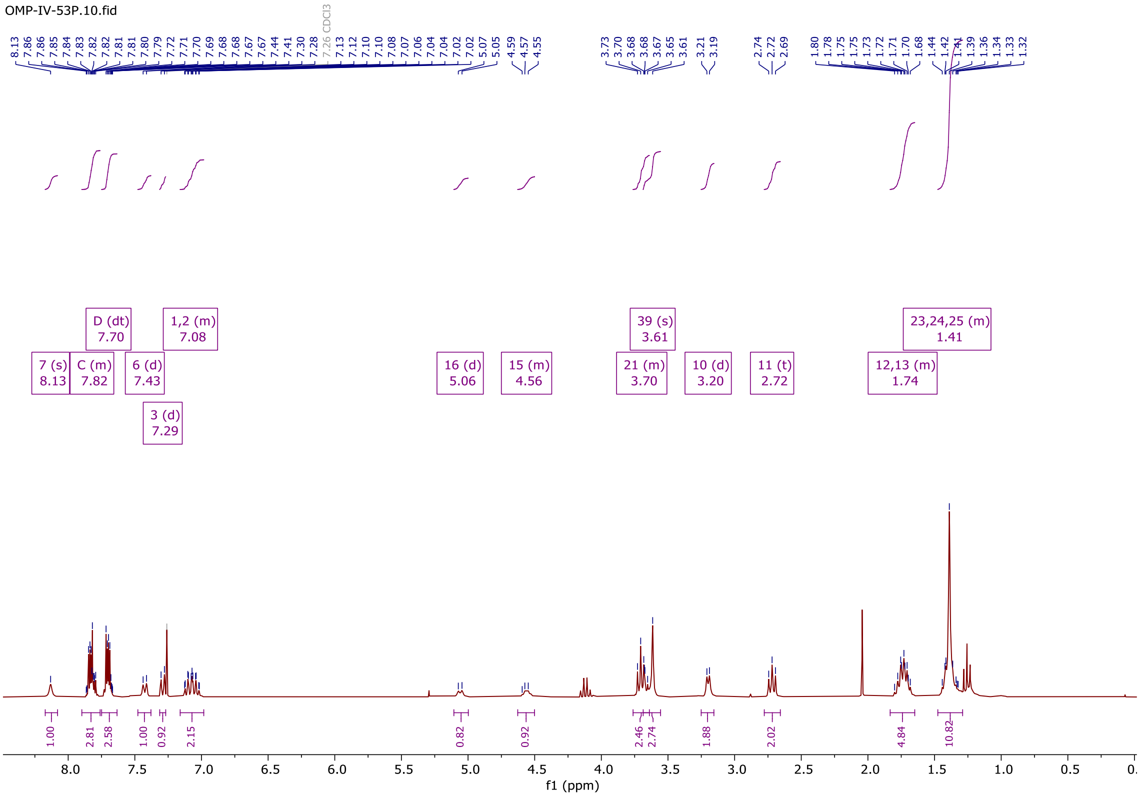

^{13}C NMR

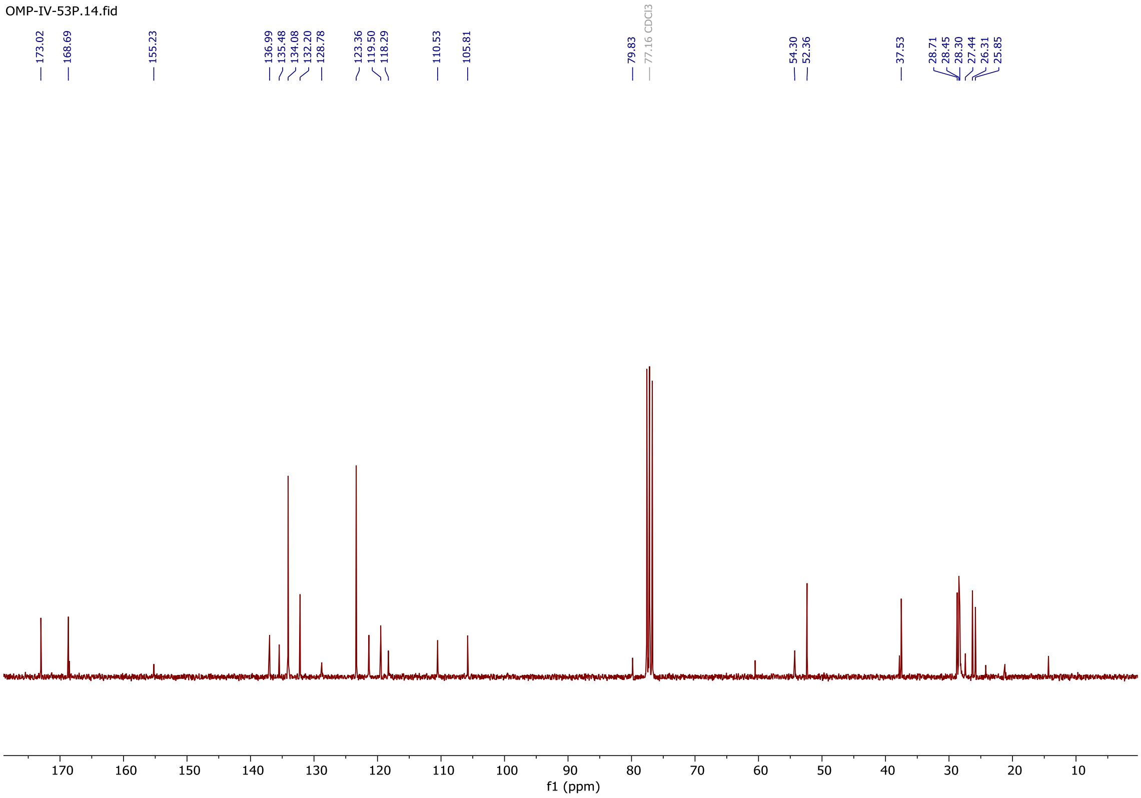

Intermediate 10

^{1}H NMR

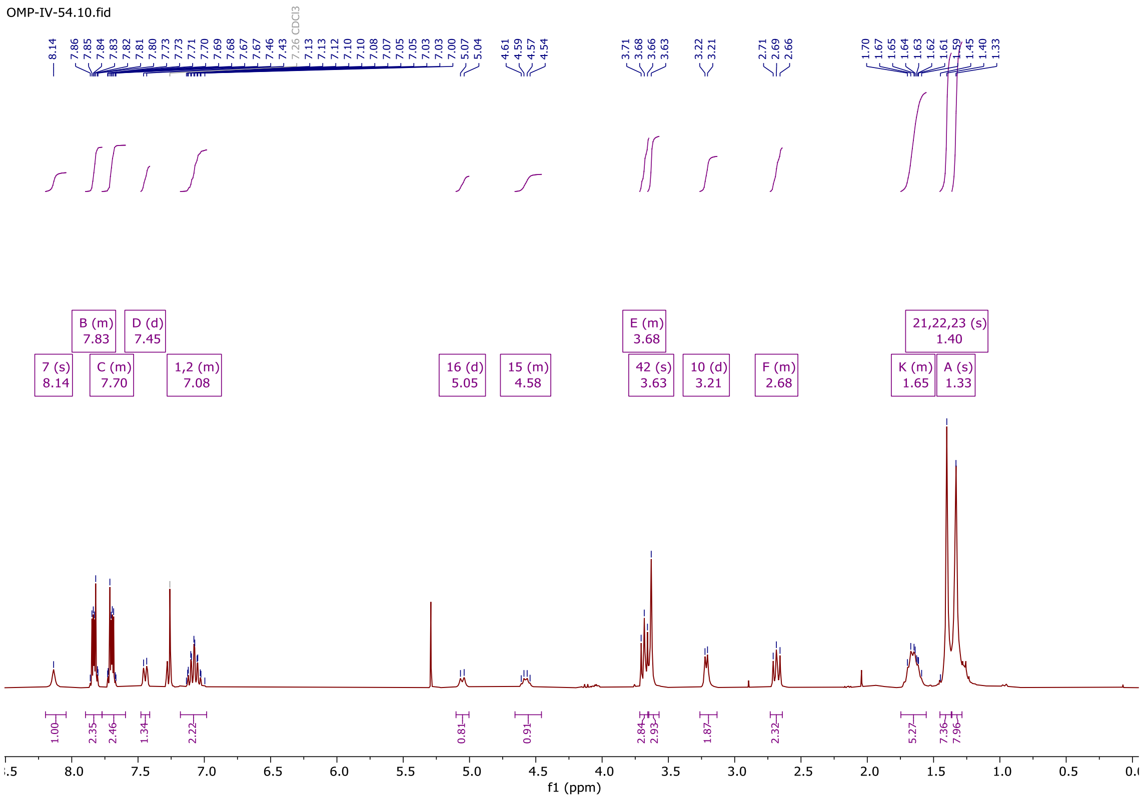

^{13}C NMR

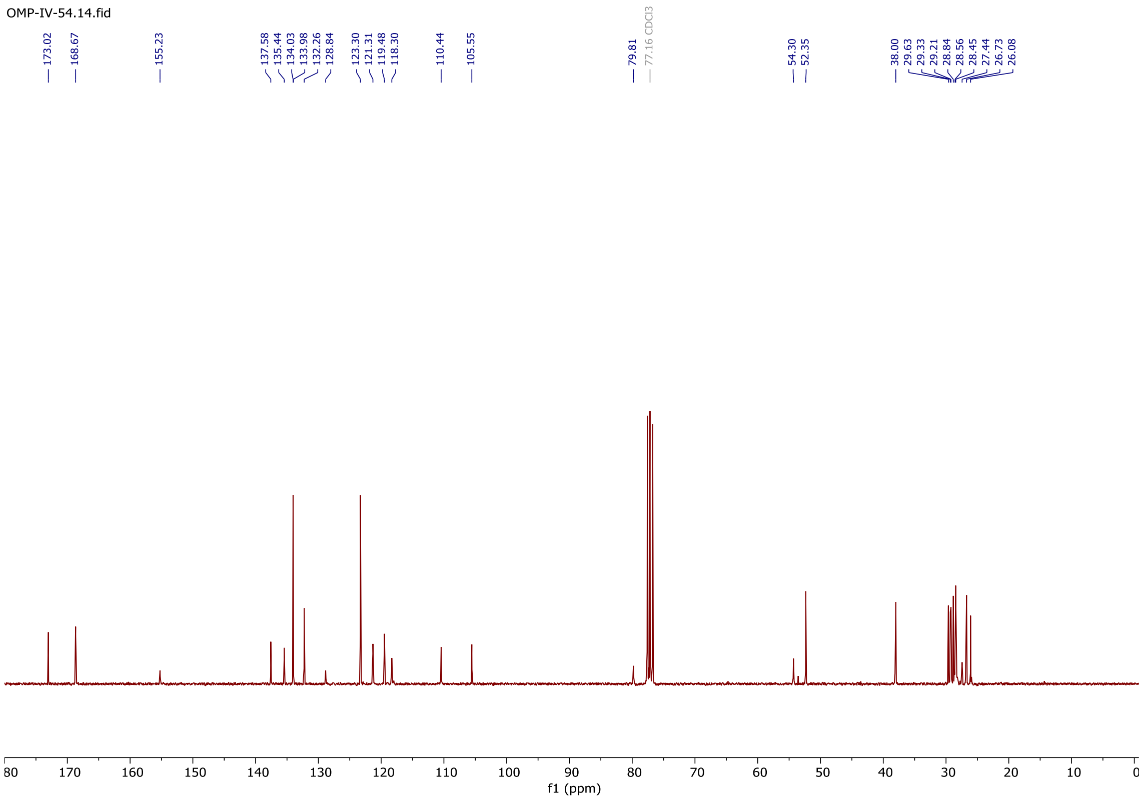

Intermediate 14

^{1}H NMR

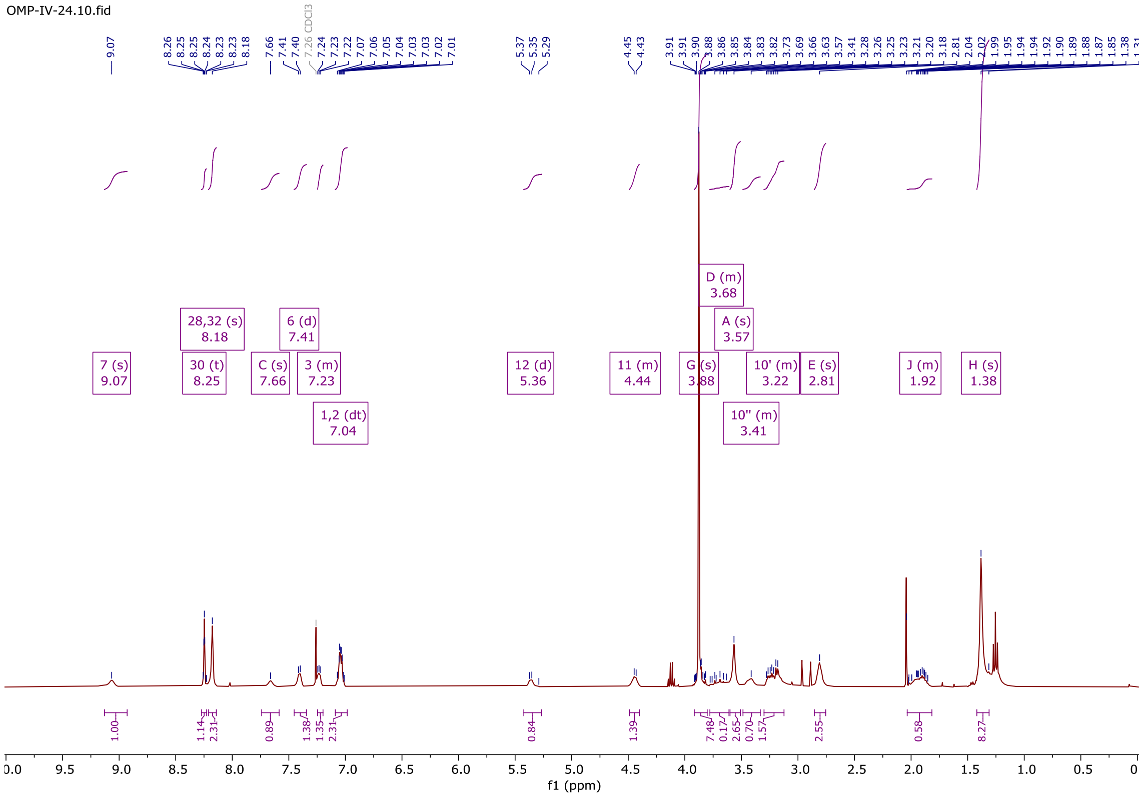

^{13}C NMR

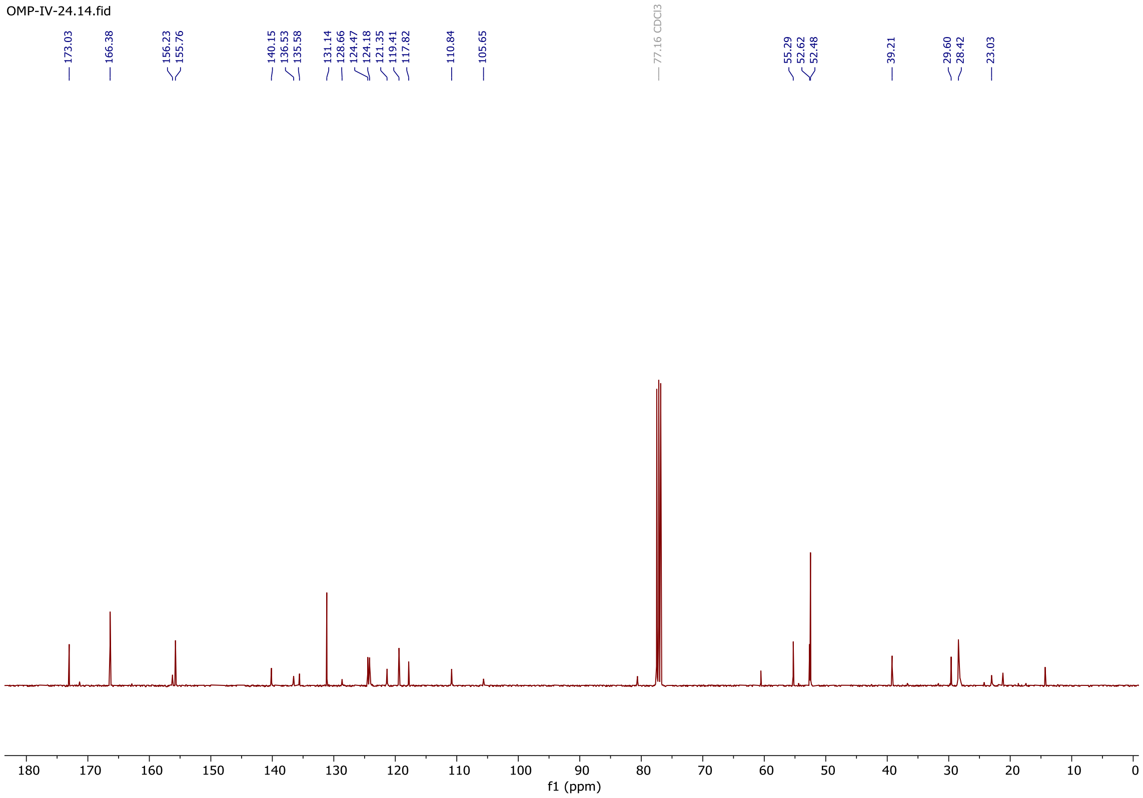

Intermediate 15

^{1}H NMR

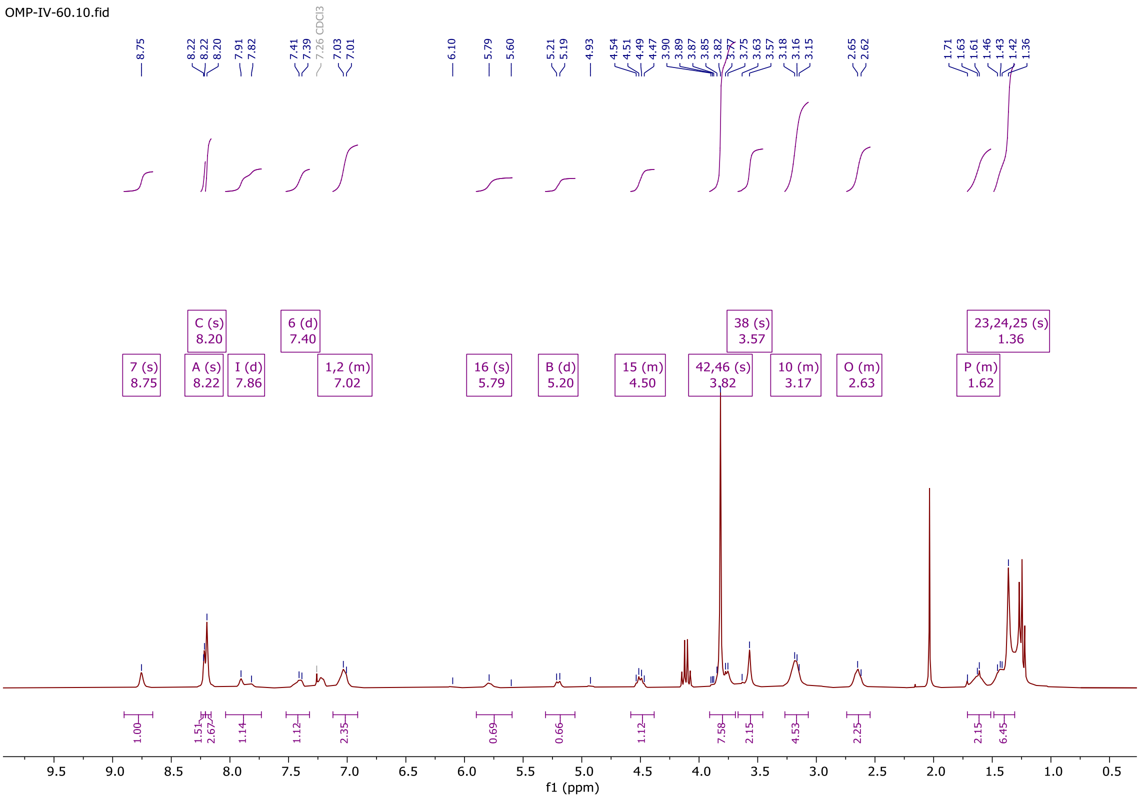

^{13}C NMR

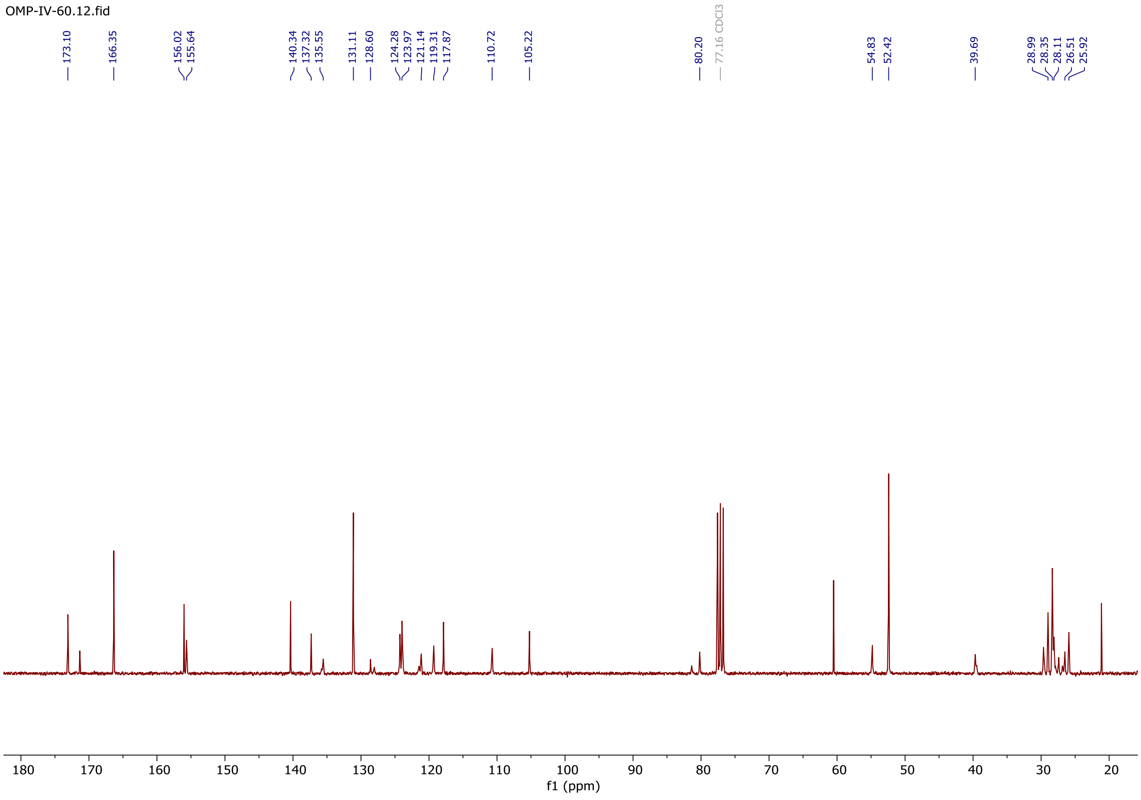

Intermediate 16

^{1}H NMR

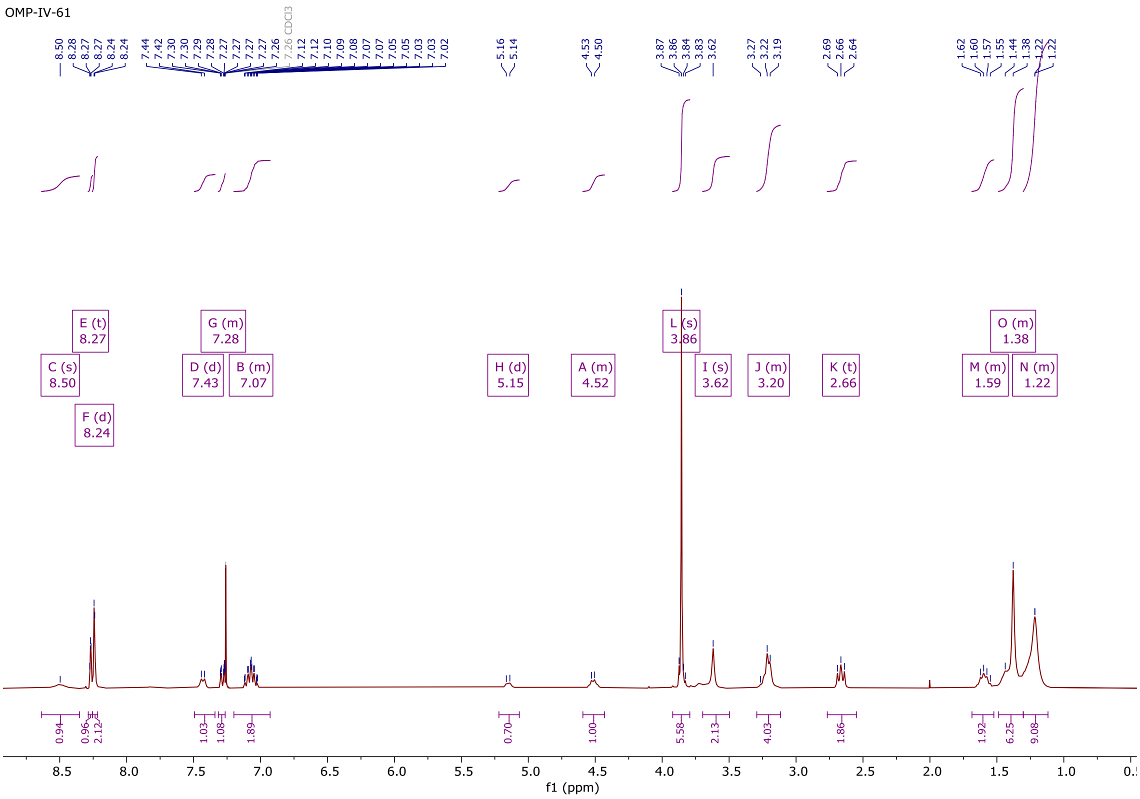

^{13}C NMR

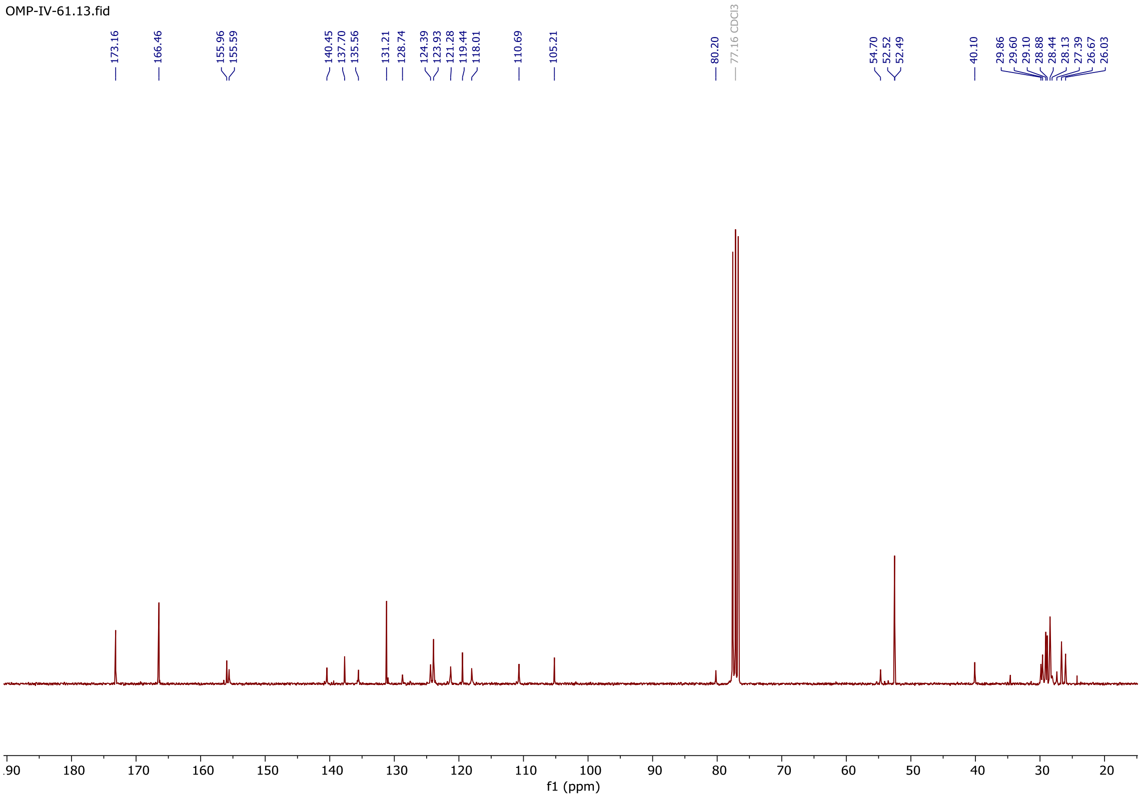

Tetramer 20

¹H NMR

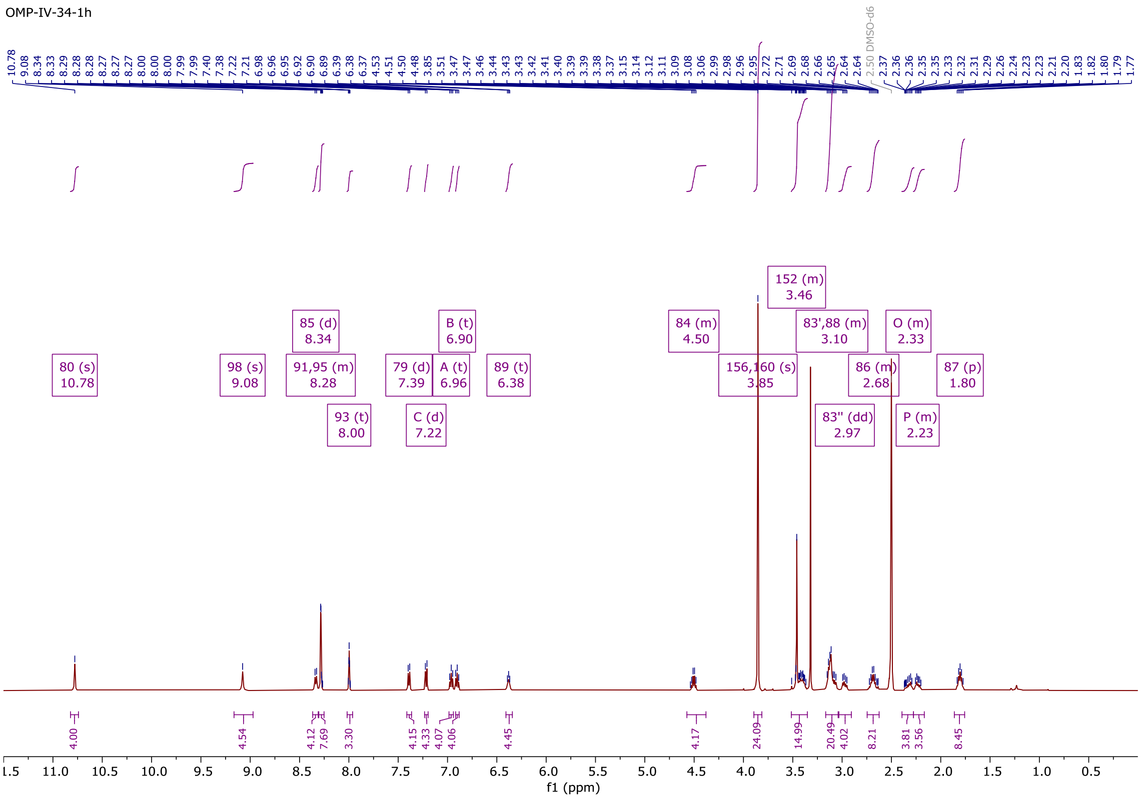

¹³C NMR

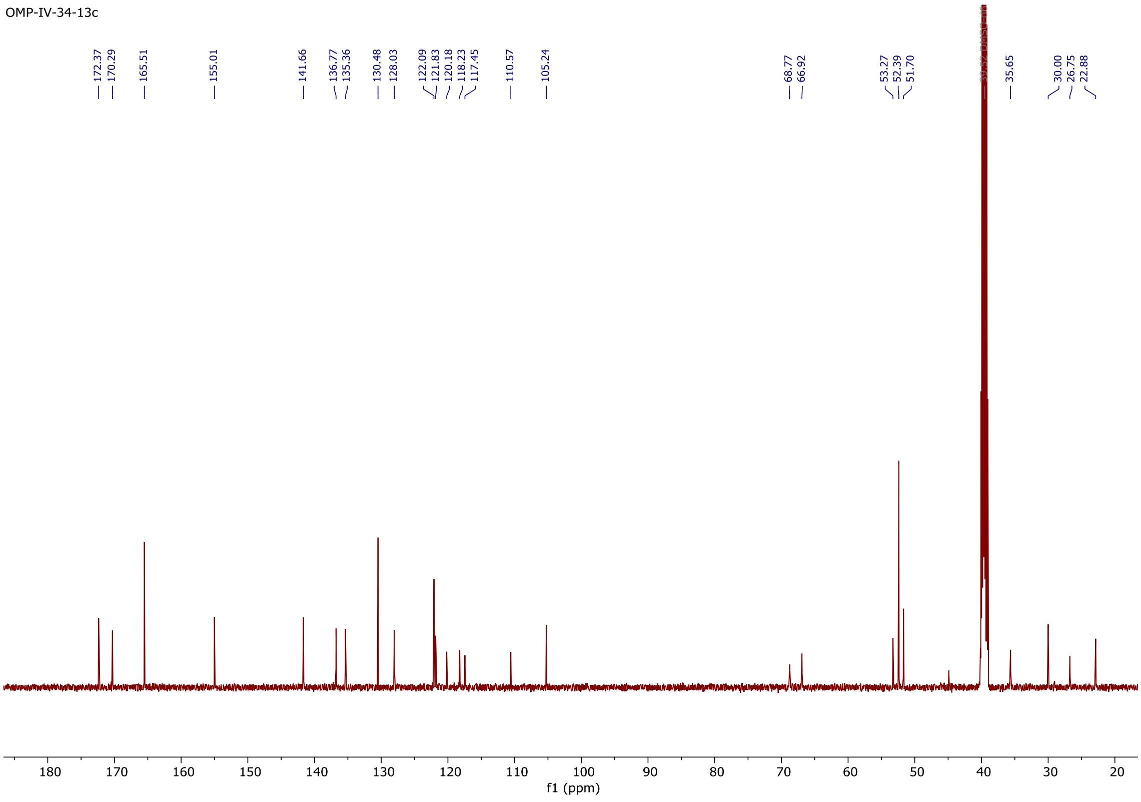

Tetramer 21

^{1}H NMR

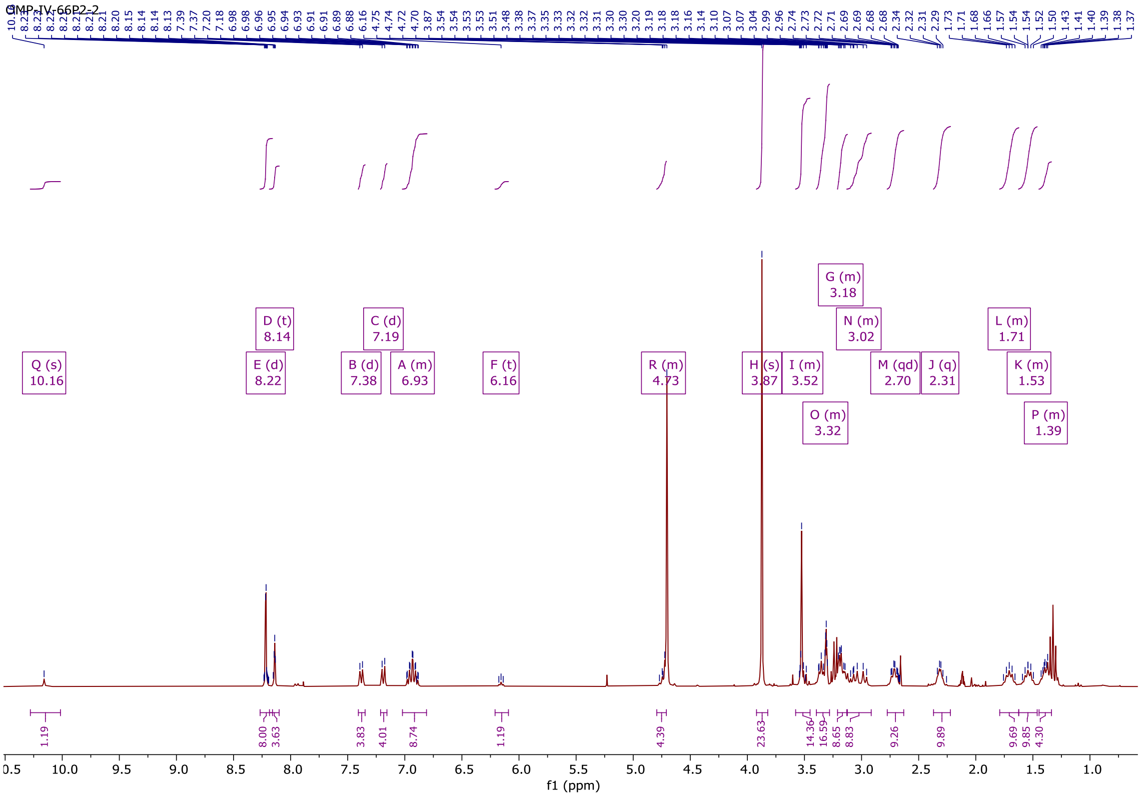

^{13}C NMR

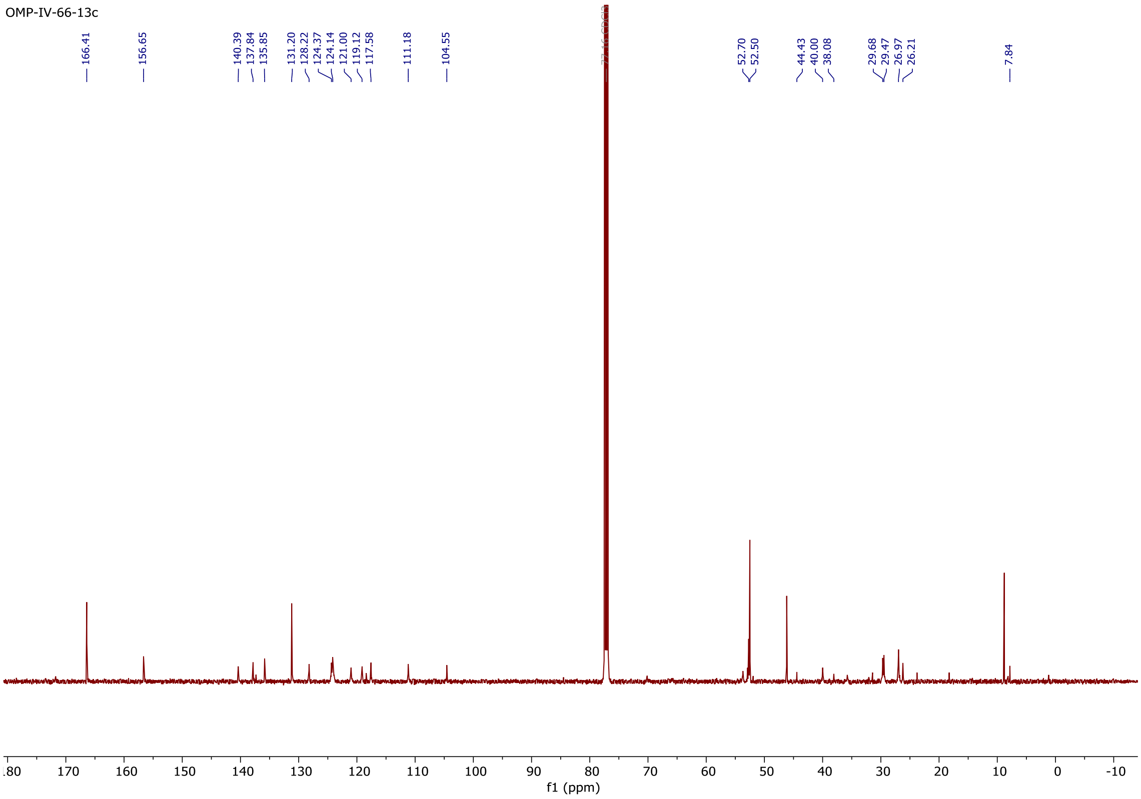

Tetramer 22

¹H NMR

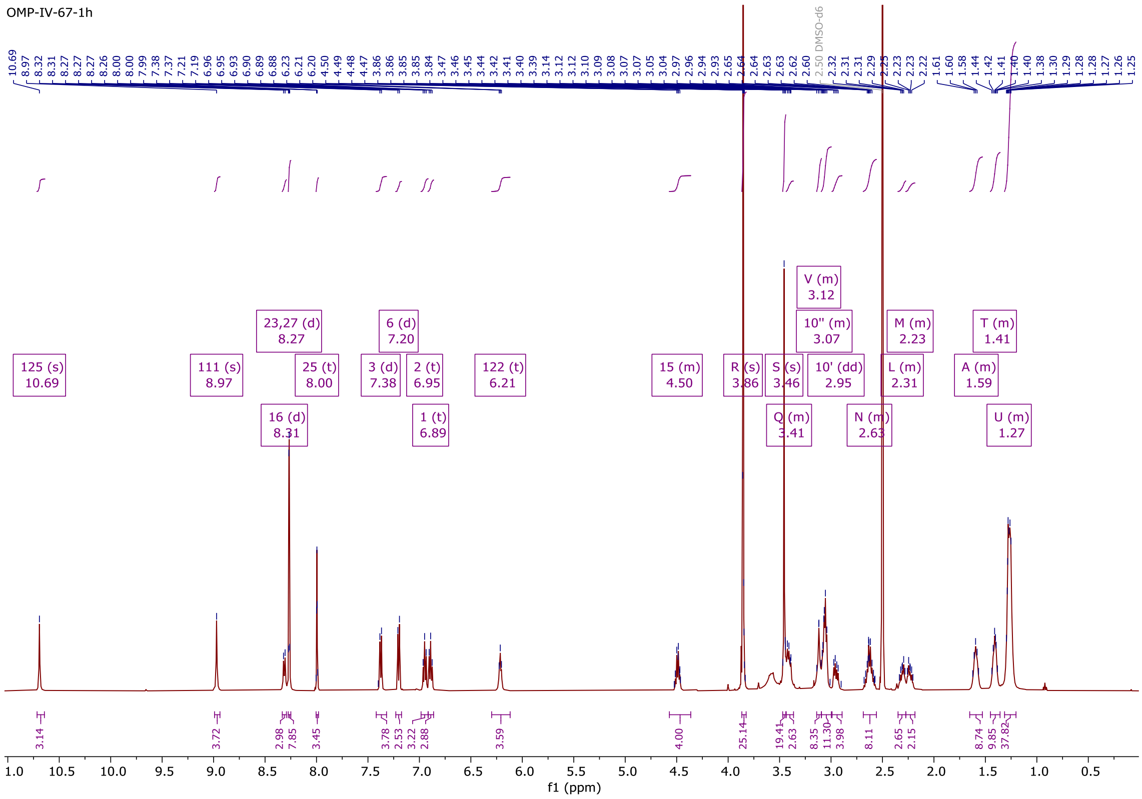

¹³C NMR

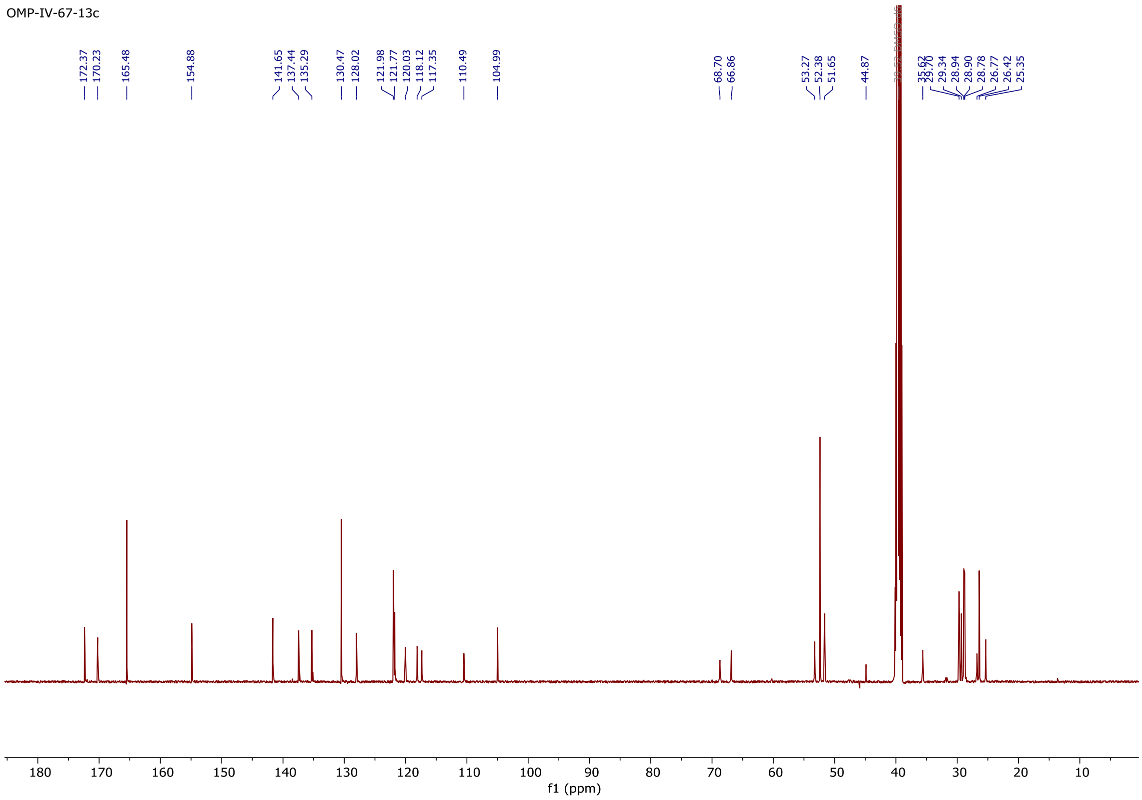

Tetramer 23

^{1}H NMR

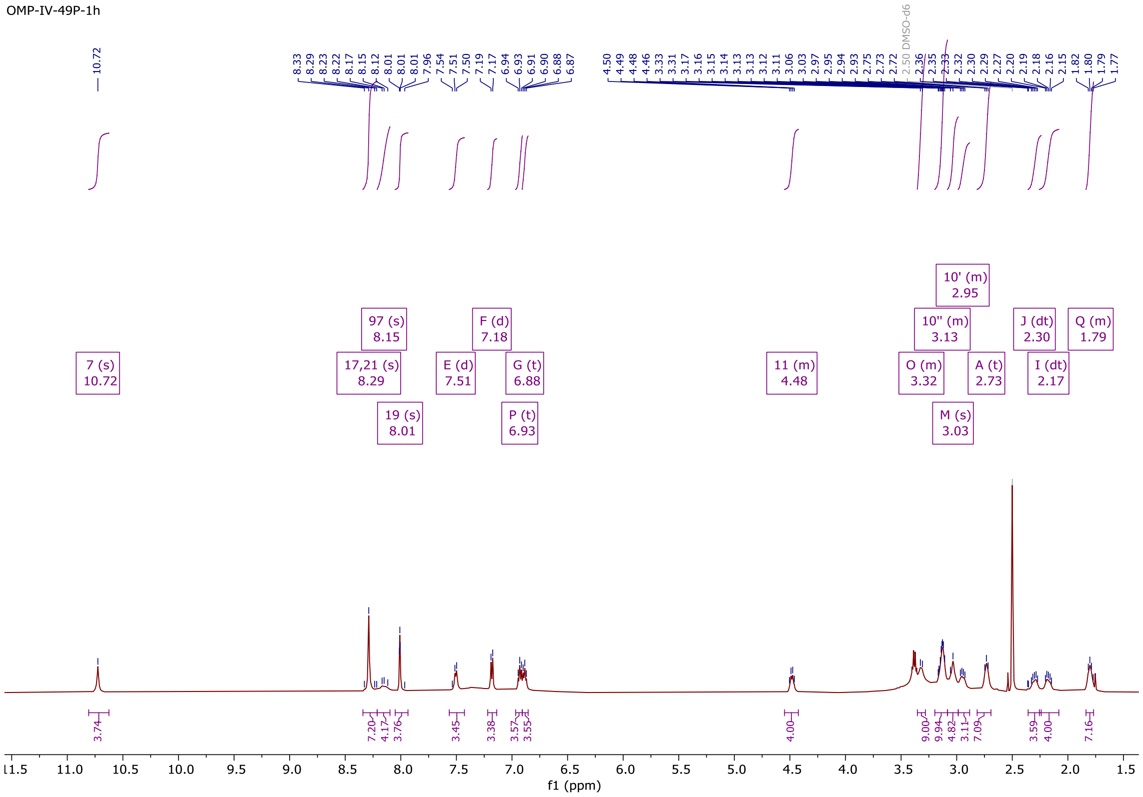

^{13}C NMR

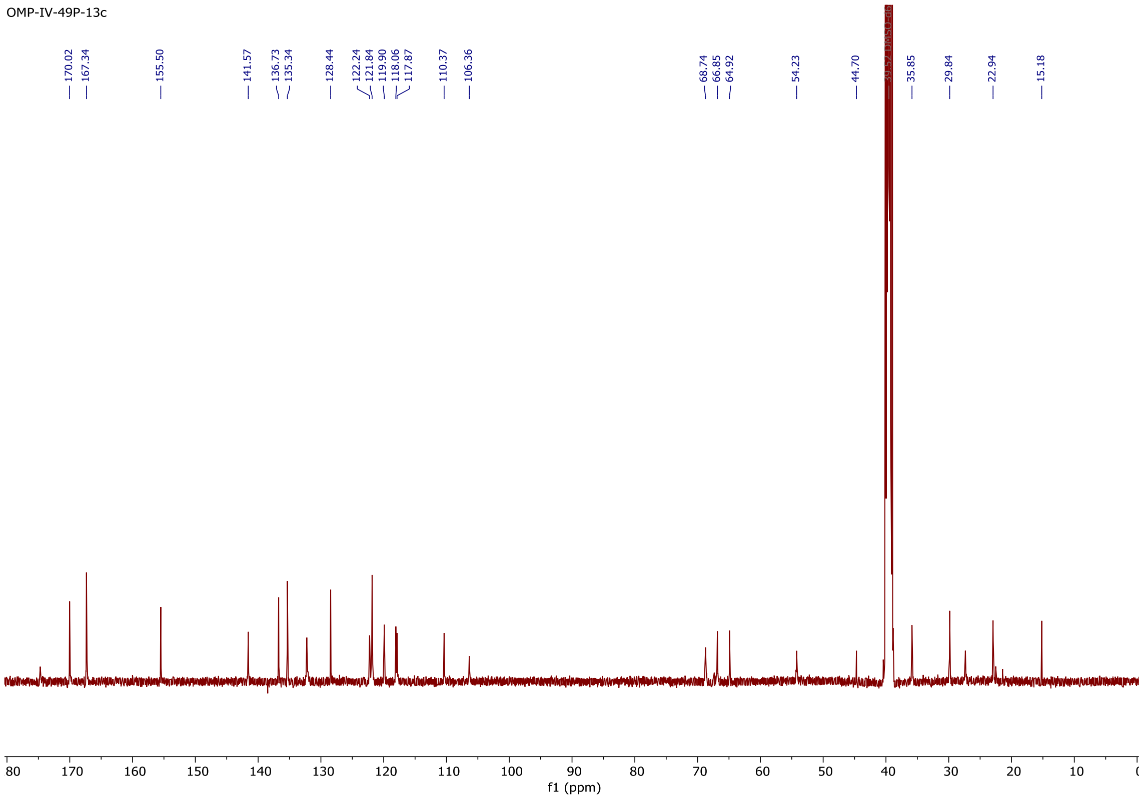

MS Spectrum

Compound Table

Compound Label	RT	Mass	Abund	Formula	Tgt Mass	Diff (ppm)
Cpd 1: C109 H116 N16 O36	0.379	2224.76703	425	C109 H116 N16 O36	2224.77381	-3.05

Compound Label	RT	Algorithm	Mass
Cpd 1: C109 H116 N16 O36	0.379	Find By Formula	2224.76703

MS Zoomed Spectrum

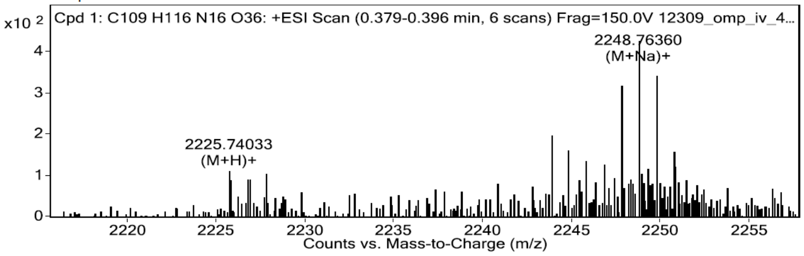

HPLC chromatogram

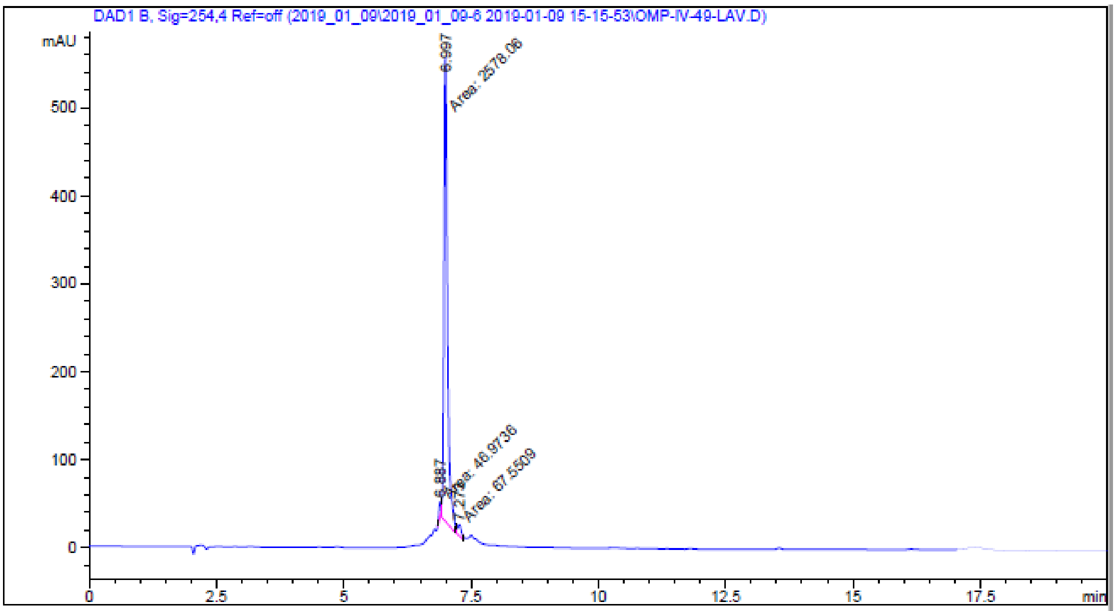


```
=====================================================================
                         Area Percent Report
=====================================================================

Sorted By             :      Signal
Multiplier:                   :      1.0000
Dilution:                     :      1.0000
Use Multiplier & Dilution Factor with ISTDs


Signal 1: DAD1 B, Sig=254,4 Ref=off

Peak RetTime Type  Width     Area      Height     Area
  #   [min]        [min]   [mAU*s]     [mAU]        %
----|-------|----|-------|----------|----------|--------|
   1   6.887 MM T  0.0461   46.97365   16.99987   1.7446
   2   6.997 MM T  0.0809 2578.06323  531.17651  95.7467
   3   7.273 MM T  0.0845   67.55088   13.31849   2.5088
```

Tetramer 24

¹H NMR

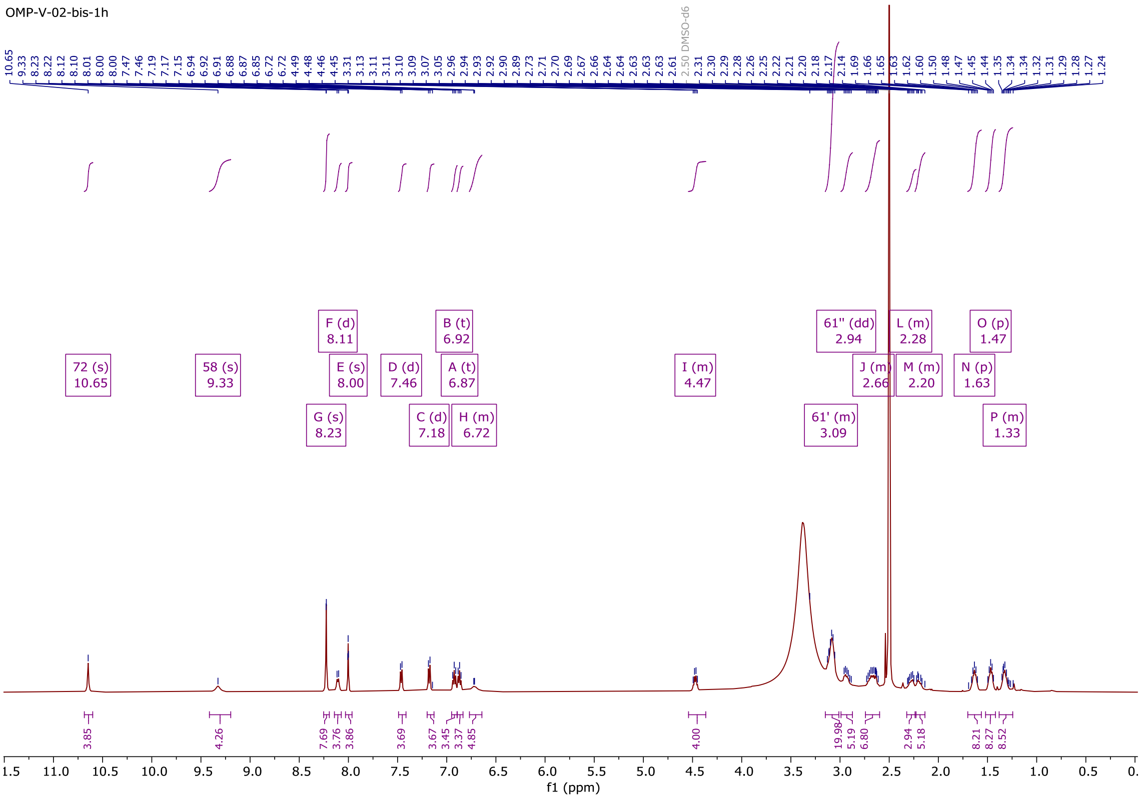

¹³C NMR

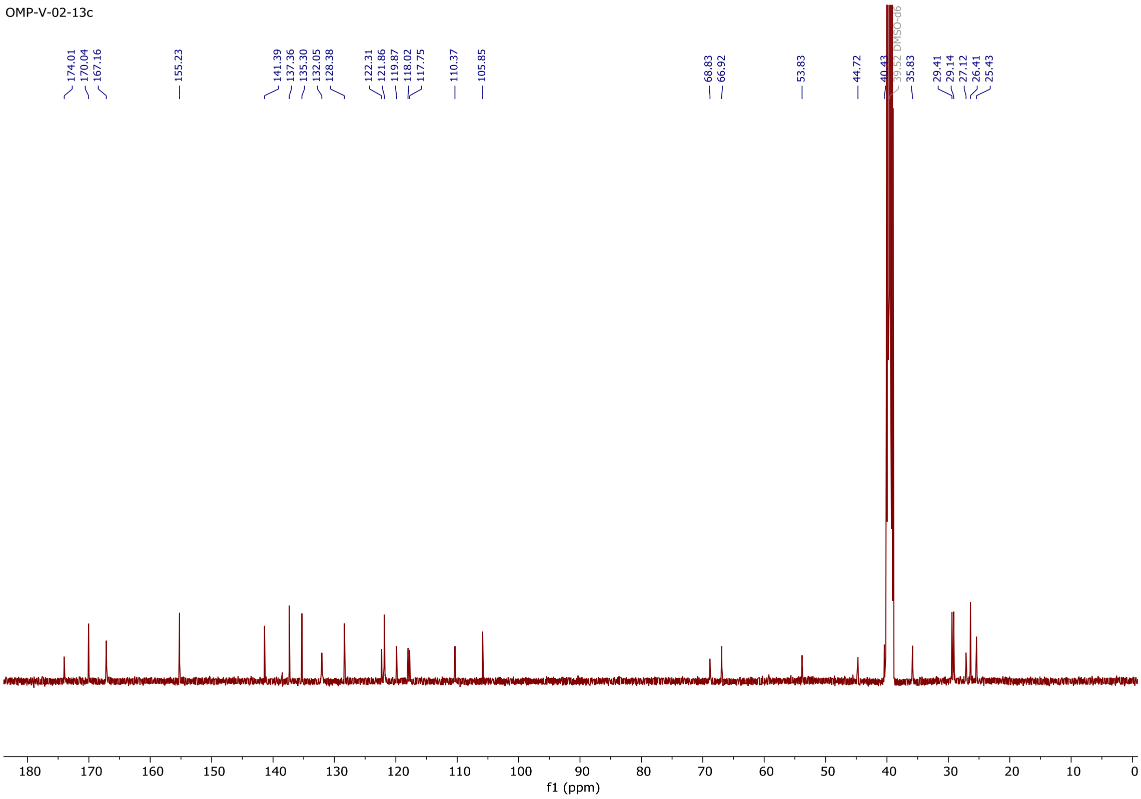

Tetramer 25

^{1}H NMR

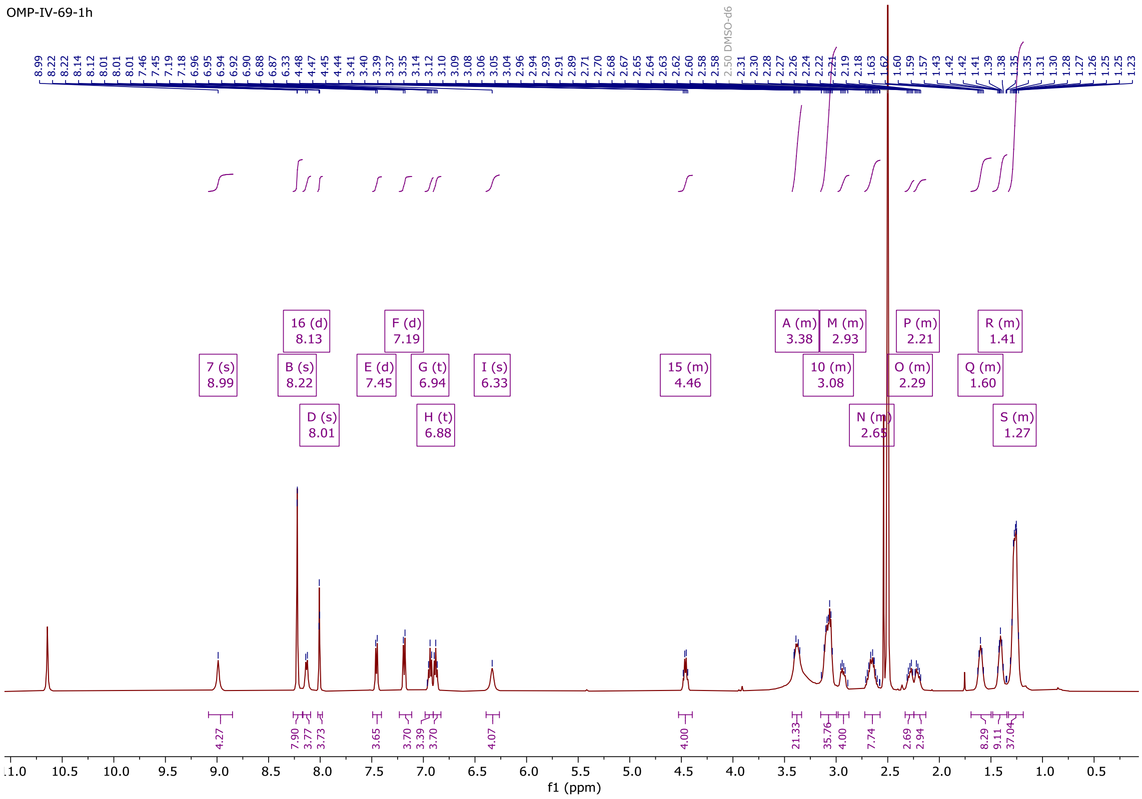

^{13}C NMR

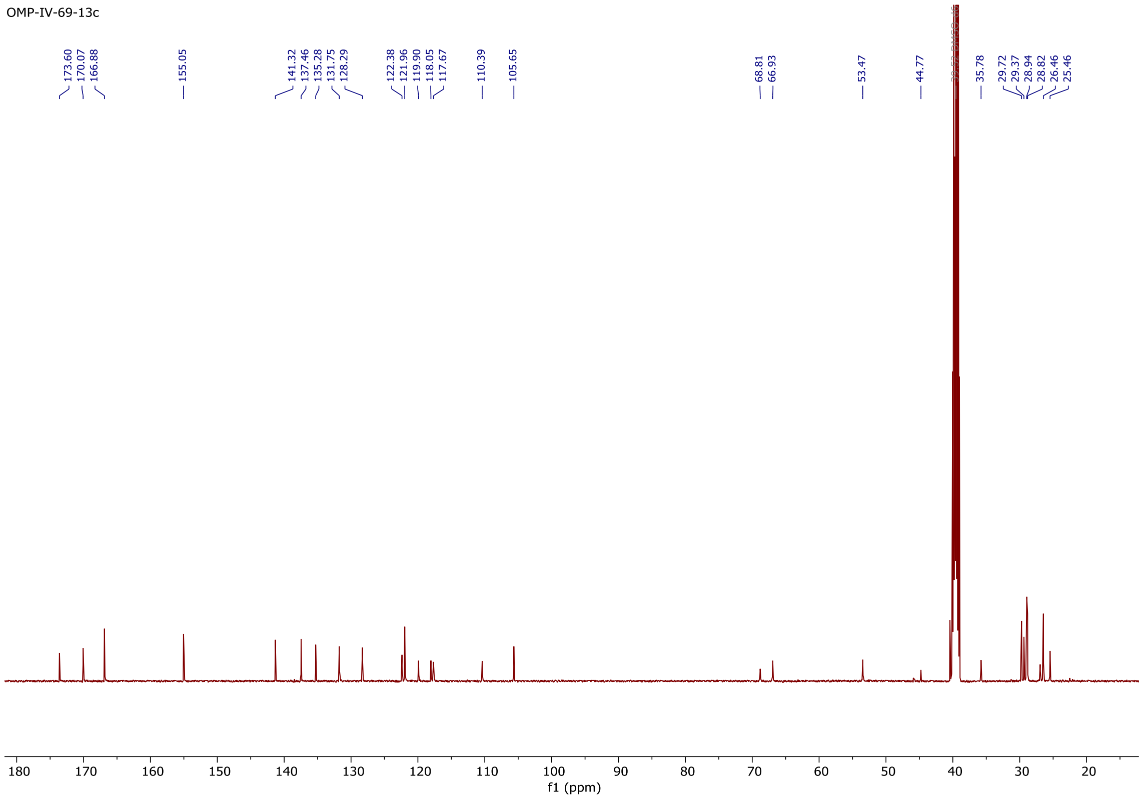

MS Spectrum

Compound Table

Compound Label	RT	Mass	Abund	Formula	Tgt Mass	Diff (ppm)
Cpd 1: C129 H156 N16 O36	0.454	2505.08475	8972	C129 H156 N16 O36	2505.08682	-0.82

Compound Label	RT	Algorithm	Mass
Cpd 1: C129 H156 N16 O36	0.454	Find By Formula	2505.08475

MS Zoomed Spectrum

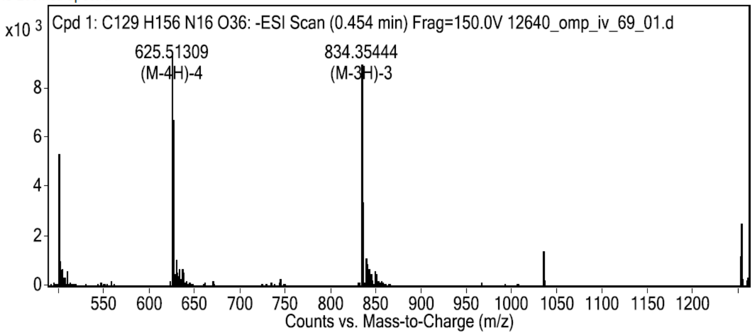

HPLC chromatogram

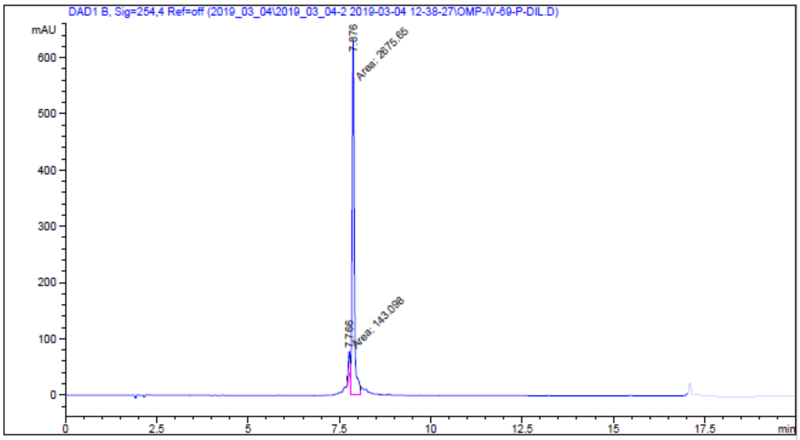


```
=====================================================================
                        Area Percent Report
=====================================================================

Sorted By             :      Signal
Multiplier:                   :      1.0000
Dilution:                     :      1.0000
Use Multiplier & Dilution Factor with ISTDs


Signal 1: DAD1 B, Sig=254,4 Ref=off

Peak RetTime Type  Width     Area      Height     Area
  #   [min]        [min]   [mAU*s]     [mAU]       %
----|-------|----|-------|----------|----------|--------|
   1   7.766 MM T  0.0514  143.09801   46.40469   5.0766
   2   7.876 MM T  0.0702 2675.65405  635.17175  94.9234

Totals :                  2818.75206  681.57644
```

Intermediate 26

^{1}H NMR

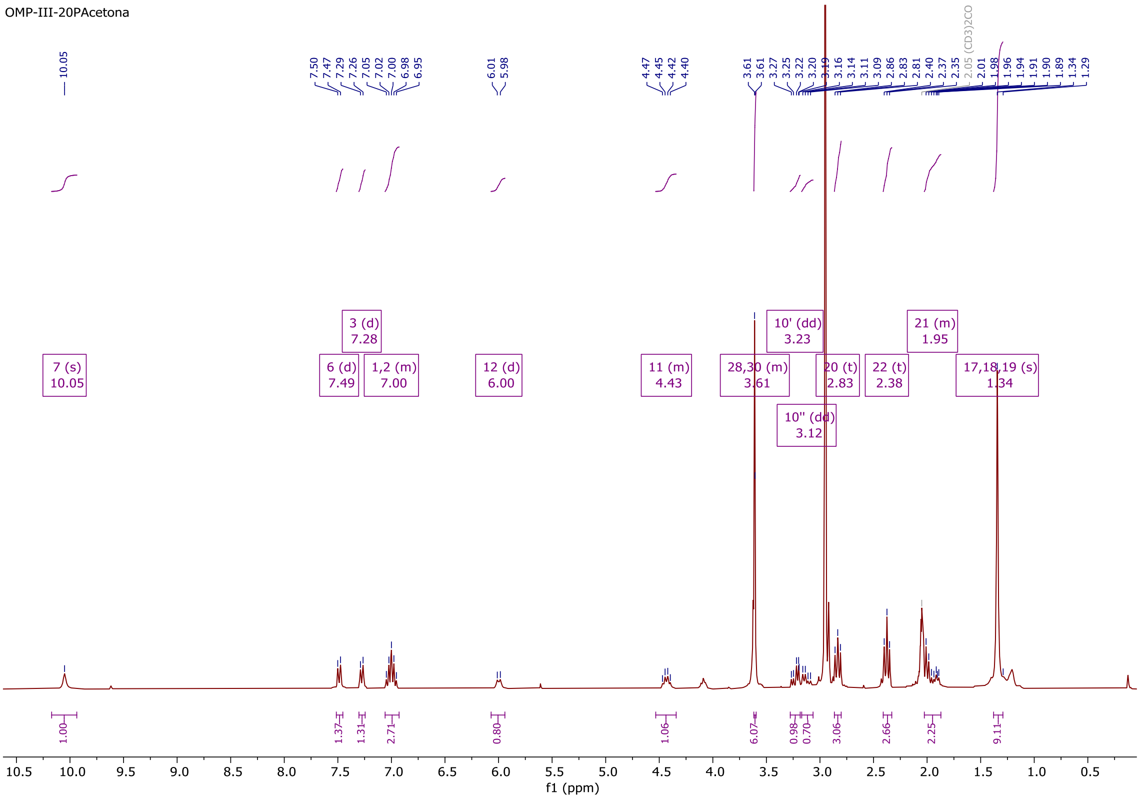

^{13}C NMR

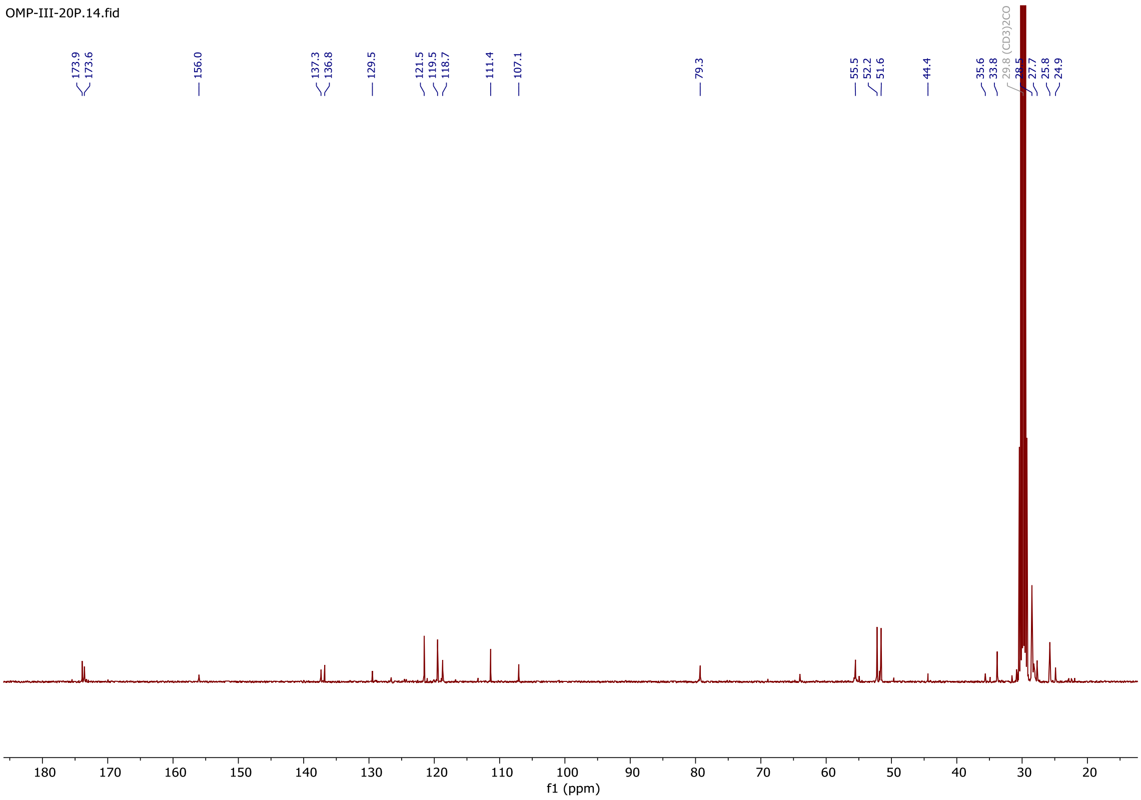

Intermediate 27

¹H NMR

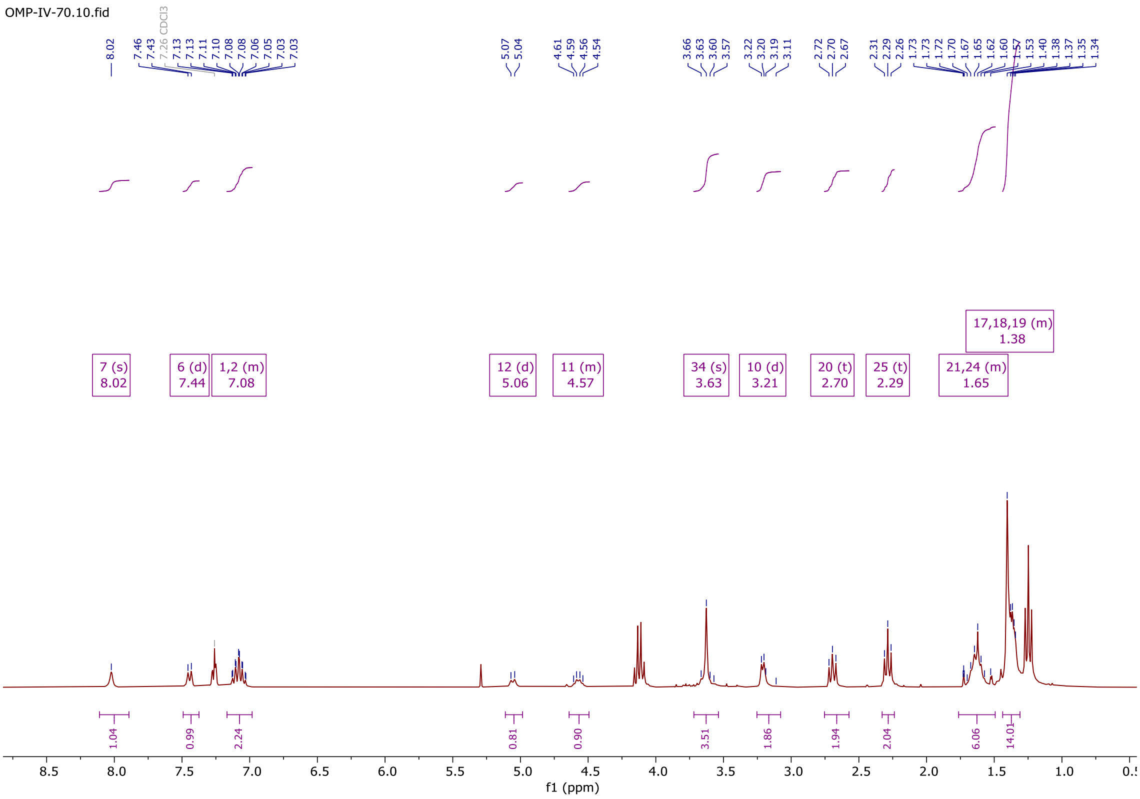

¹³C NMR

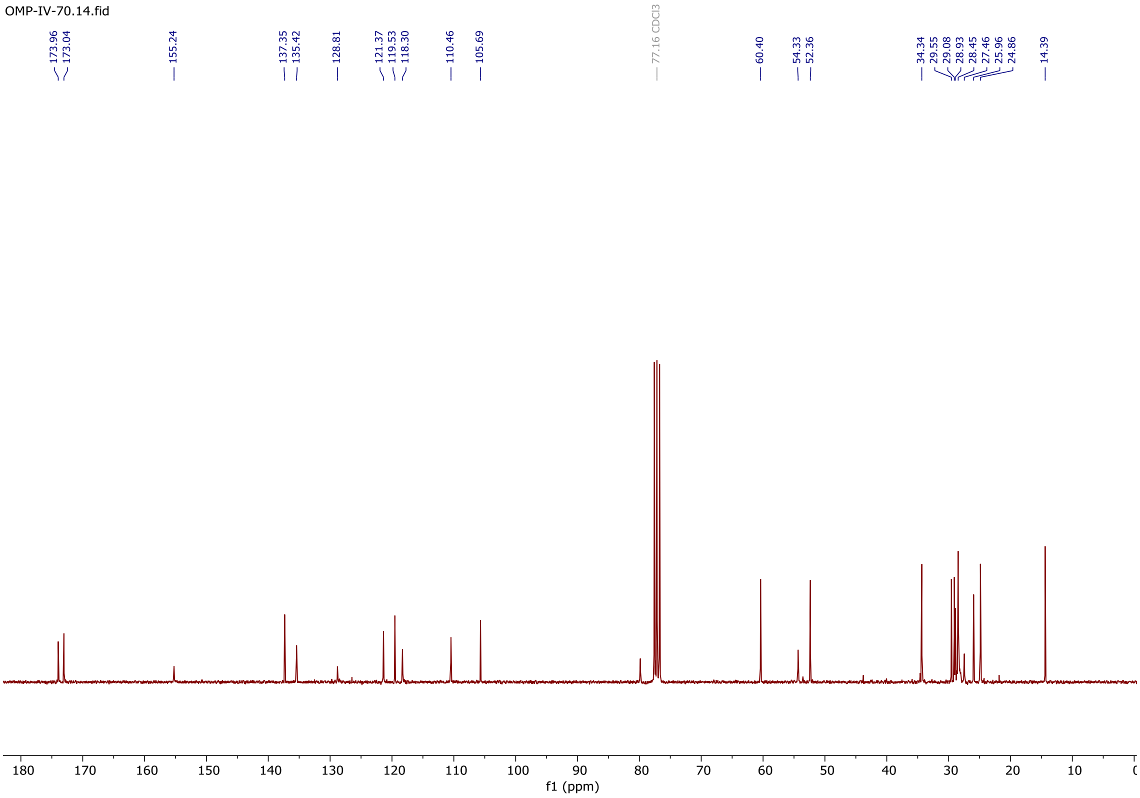

Tetramer 30

¹H NMR

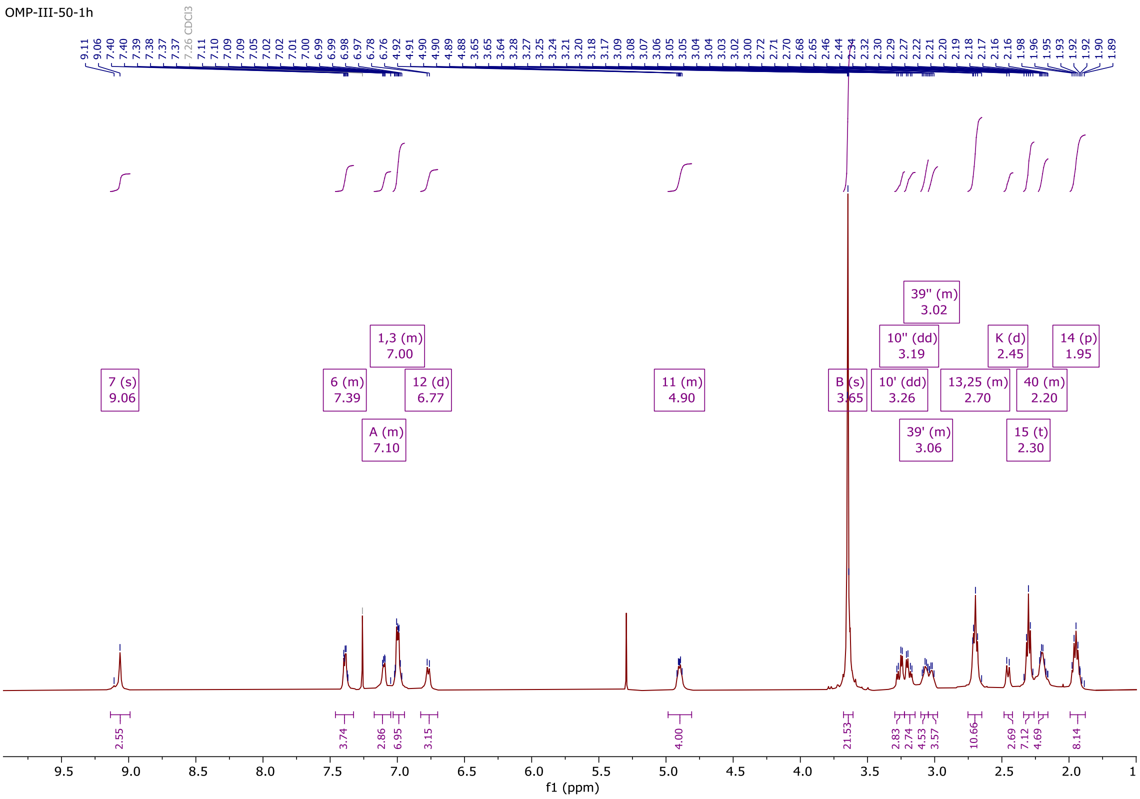

¹³C NMR

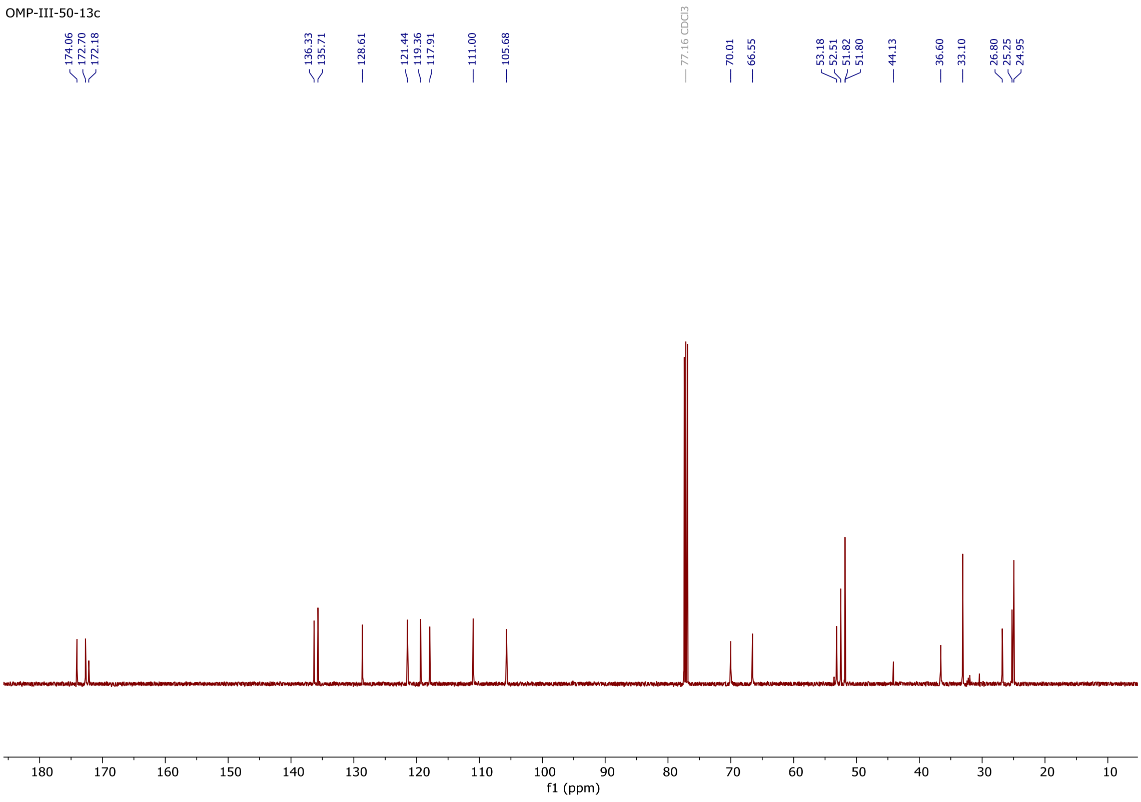

Tetramer 31

^{1}H NMR

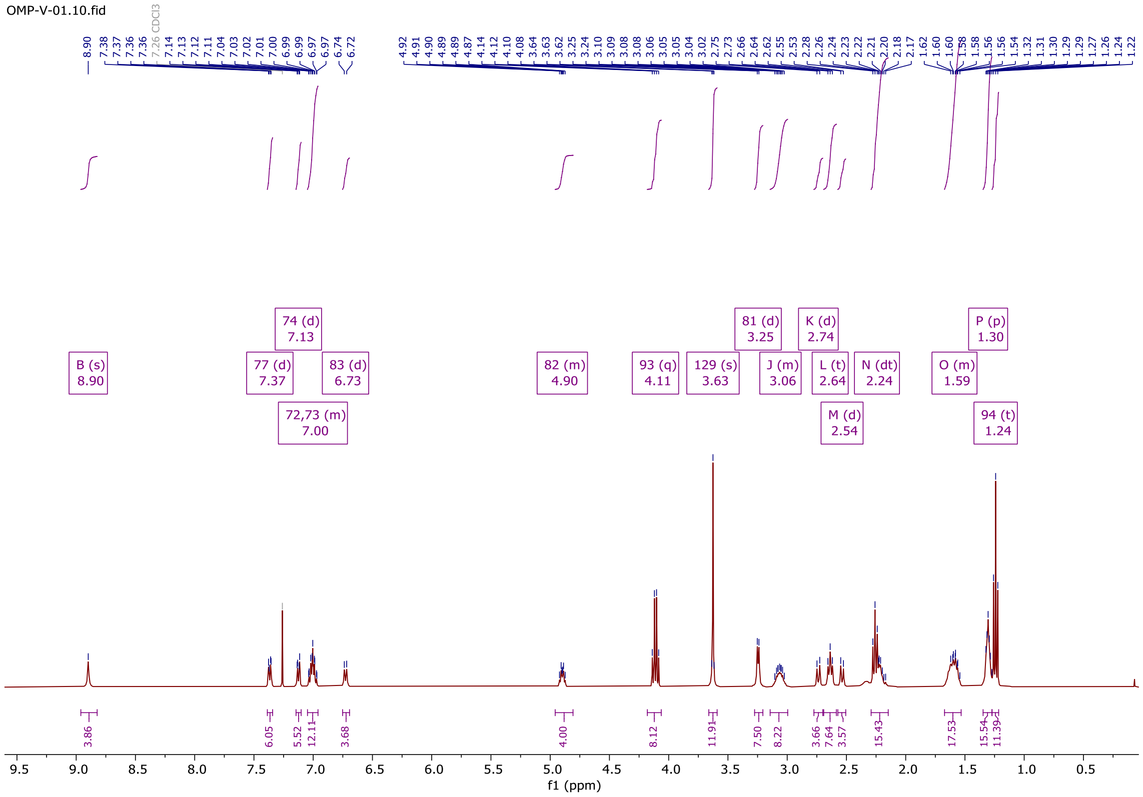

^{13}C NMR

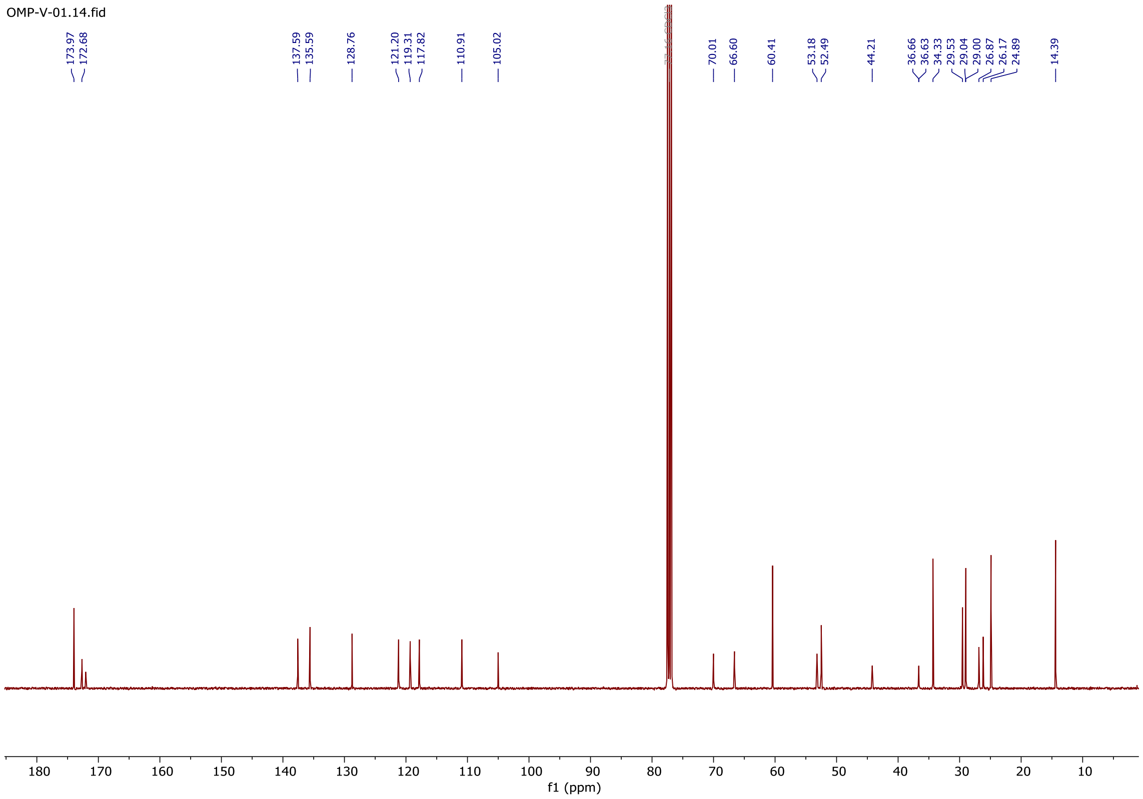

Tetramer 32

^{1}H NMR

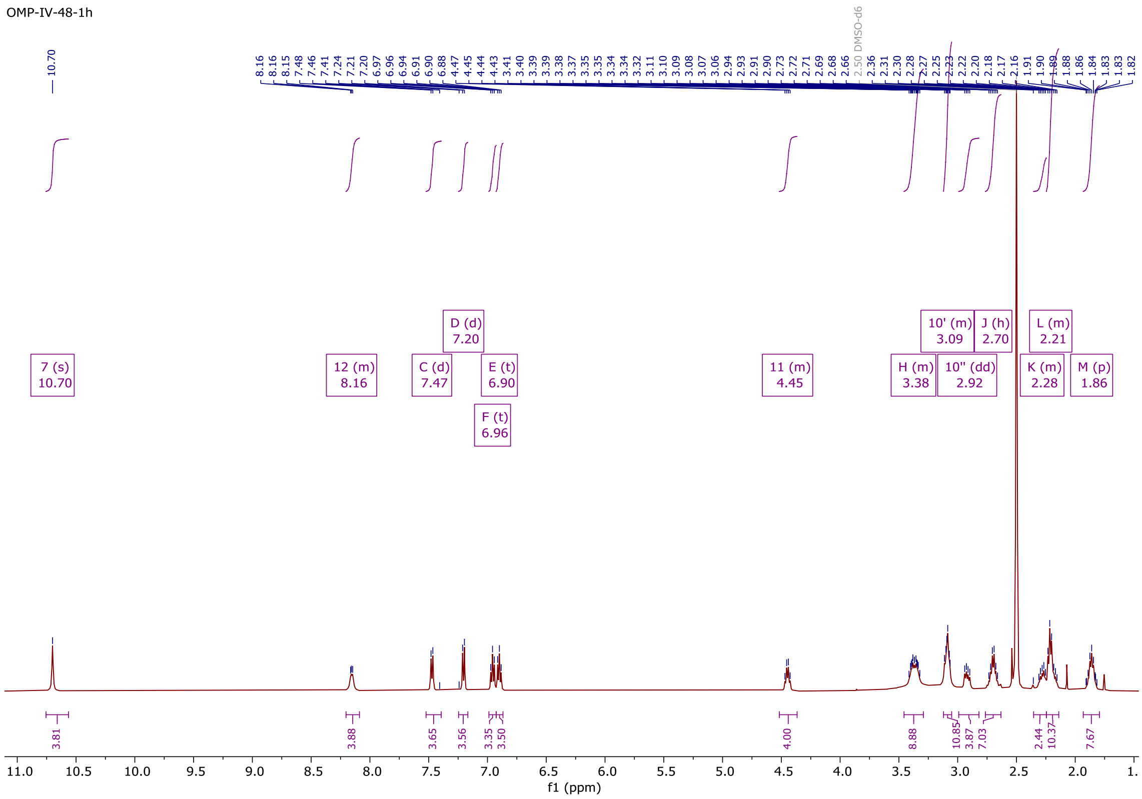

^{13}C NMR

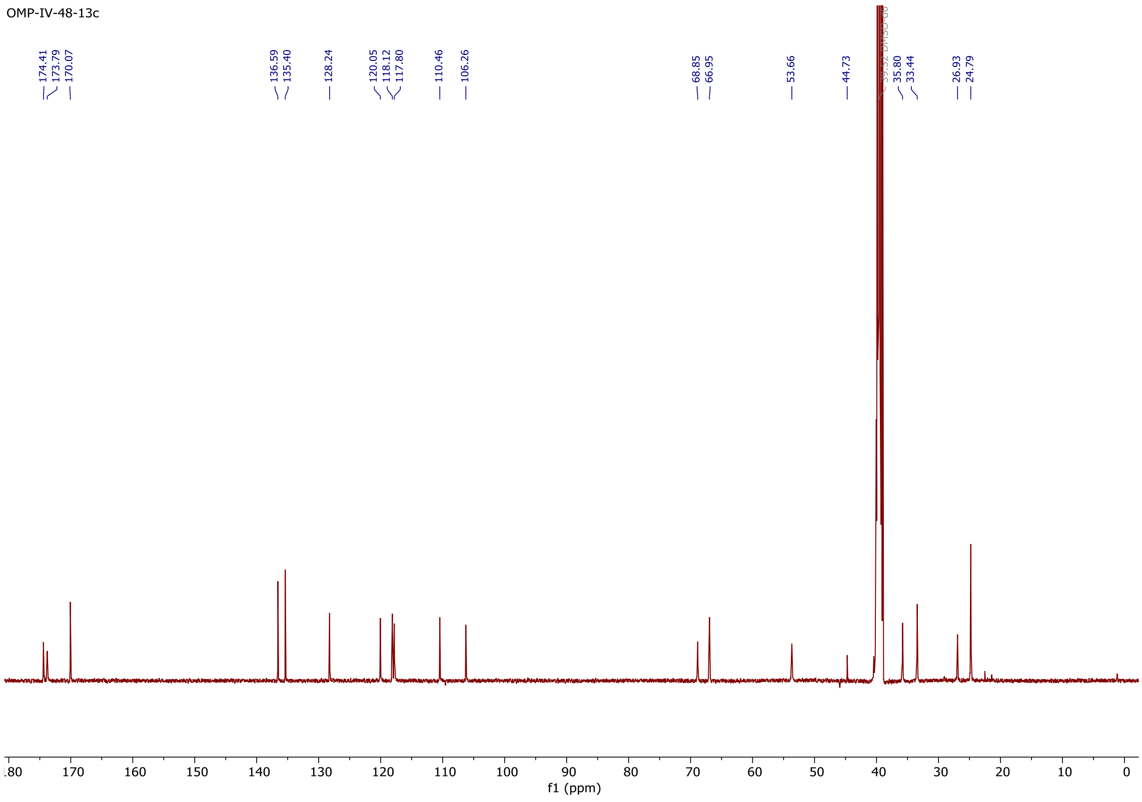

MS Spectrum

Compound Table

Compound Label	RT	Mass	Abund	Formula	Tgt Mass	Diff (ppm)
Cpd 1: C77 H92 N8 O24	0.24	1512.62337	72061	C77 H92 N8 O24	1512.62245	0.61

Compound Label	RT	Algorithm	Mass
Cpd 1: C77 H92 N8 O24	0.24	Find By Formula	1512.62337

MS Zoomed Spectrum

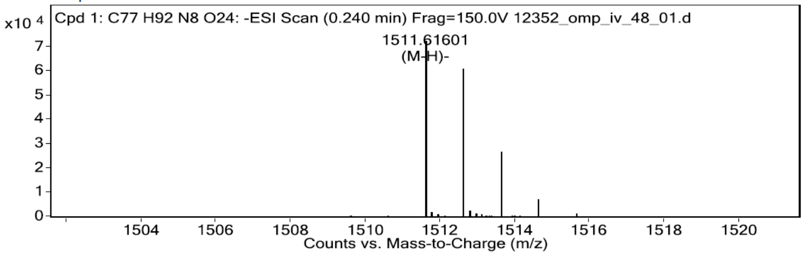

Tetramer 33

^{1}H NMR

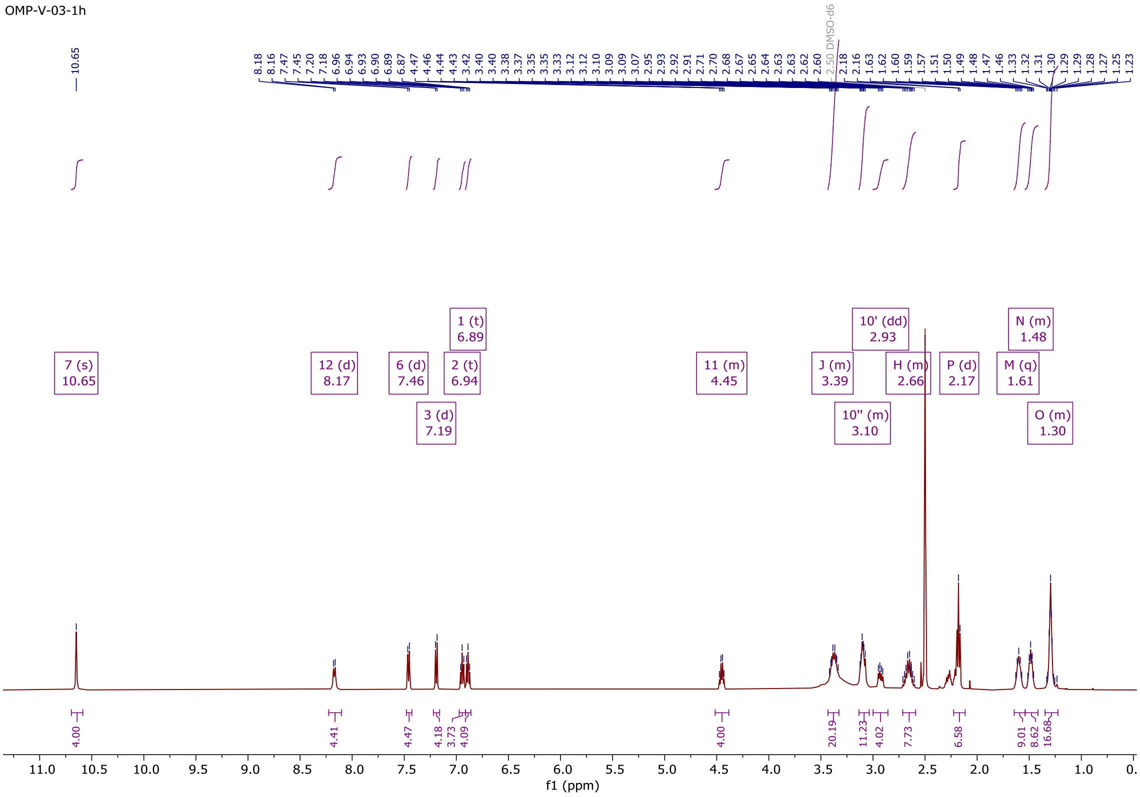

^{13}C NMR

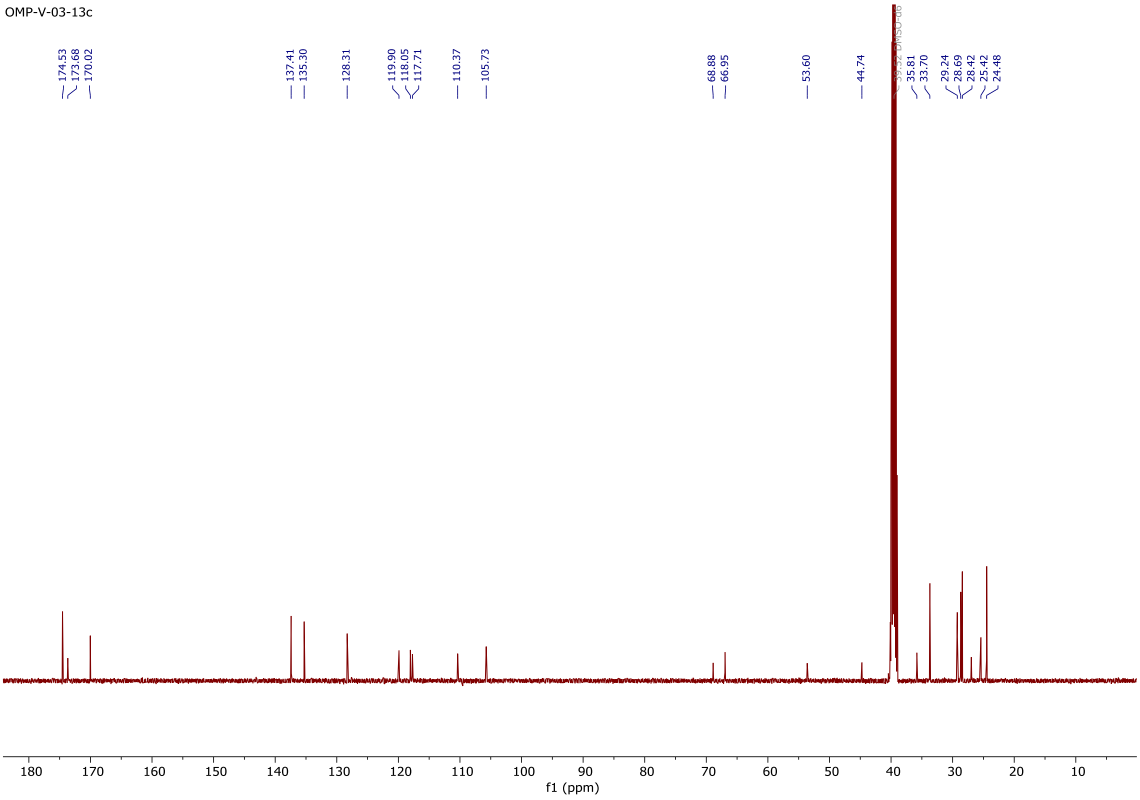

MS Spectrum

Compound Label	RT	Mass	Abund	Formula	Tgt Mass	Diff (ppm)
Cpd 1: C89 H116 N8 O24	0.267	1680.80782	31635	C89 H116 N8 O24	1680.81025	-1.45

Compound Label	RT	Algorithm	Mass
Cpd 1: C89 H116 N8 O24	0.267	Find By Formula	1680.80782

MS Zoomed Spectrum

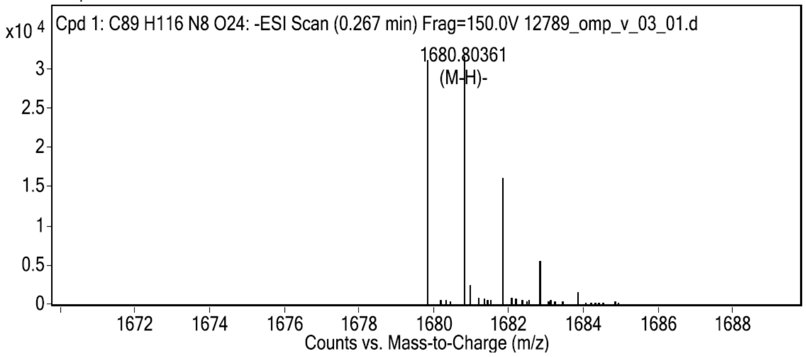

HPLC chromatogram

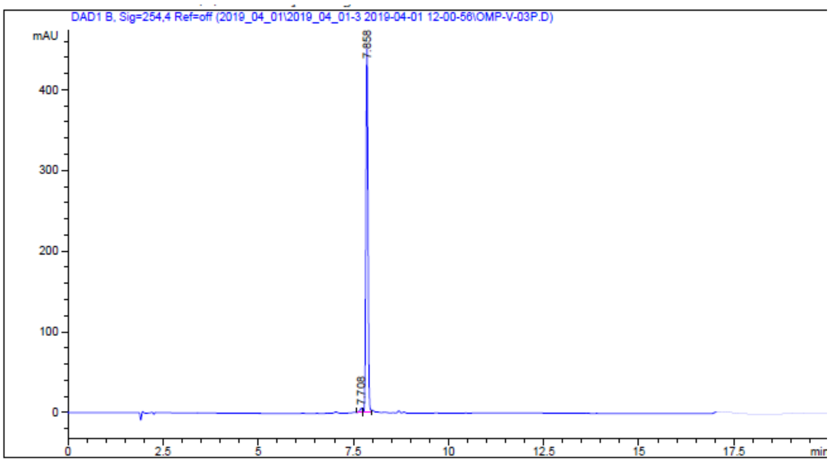

===

Area Percent Report

===

Sorted By : Signal
Multiplier: : 1.0000
Dilution: : 1.0000
Use Multiplier & Dilution Factor with ISTDs

Signal 1: DAD1 B, Sig=254,4 Ref=off

Peak #	RetTime [min]	Type	Width [min]	Area [mAU*s]	Height [mAU]	Area %
1	7.708	BV	0.0701	25.59484	5.52208	1.4588
2	7.858	VV	0.0606	1728.91431	452.49088	98.5412
Totals :				1754.50914	458.01295	

Tetramer 34

^{1}H NMR

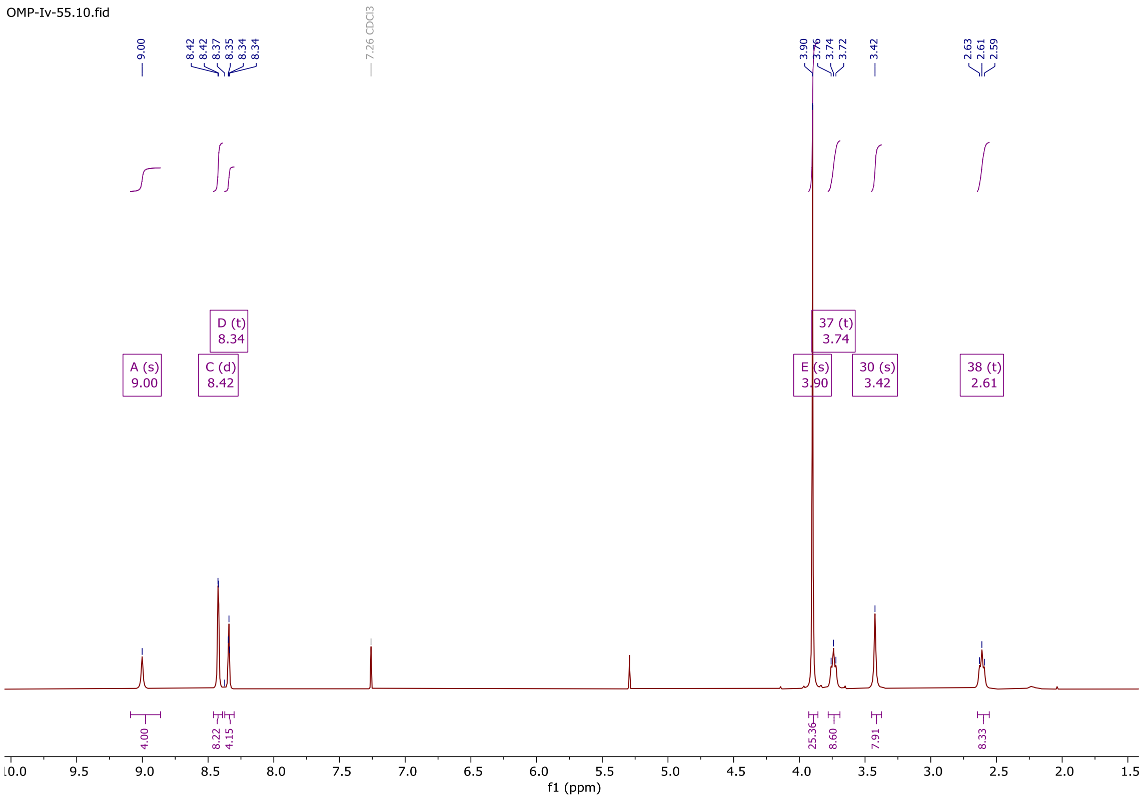

^{13}C NMR

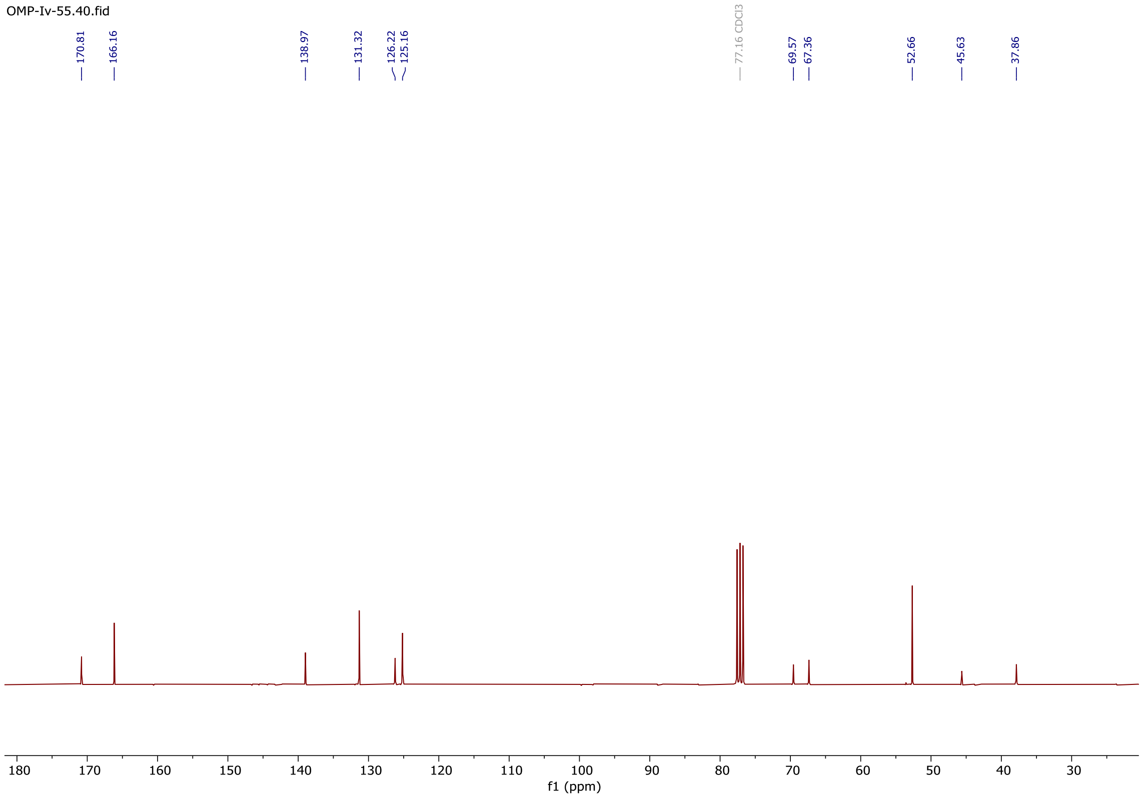

Tetramer 35

^{1}H NMR

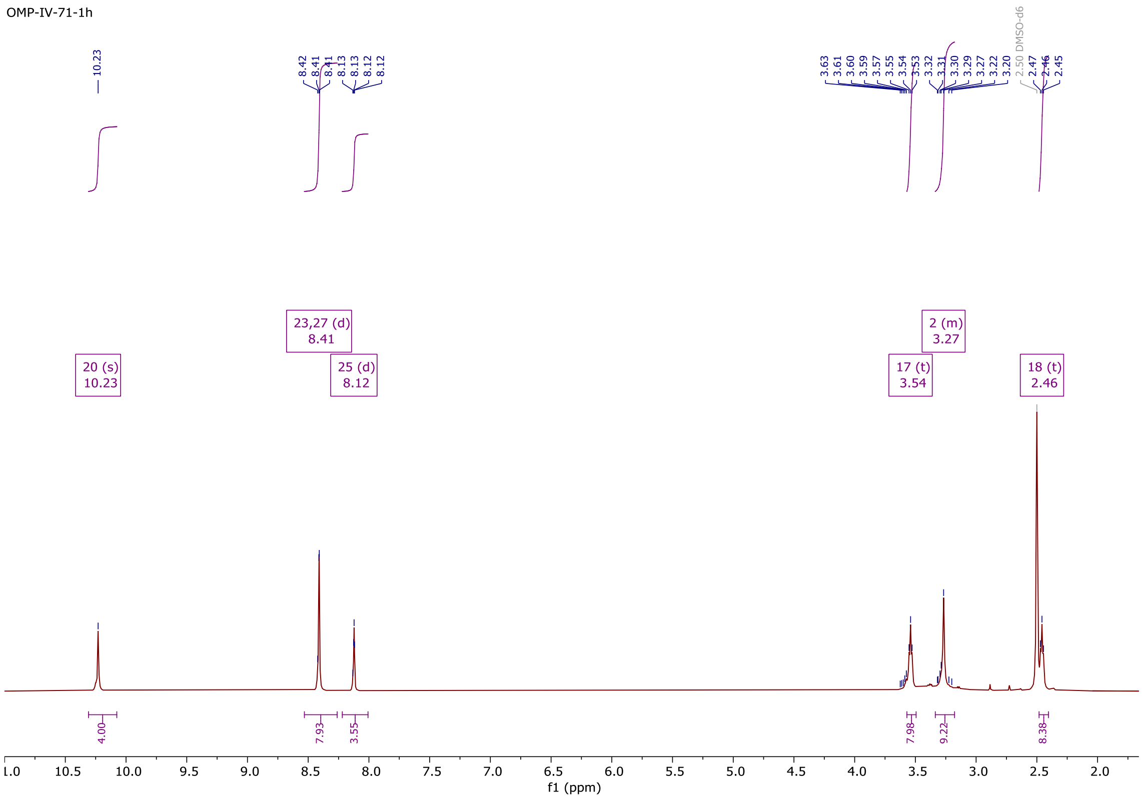

^{13}C NMR

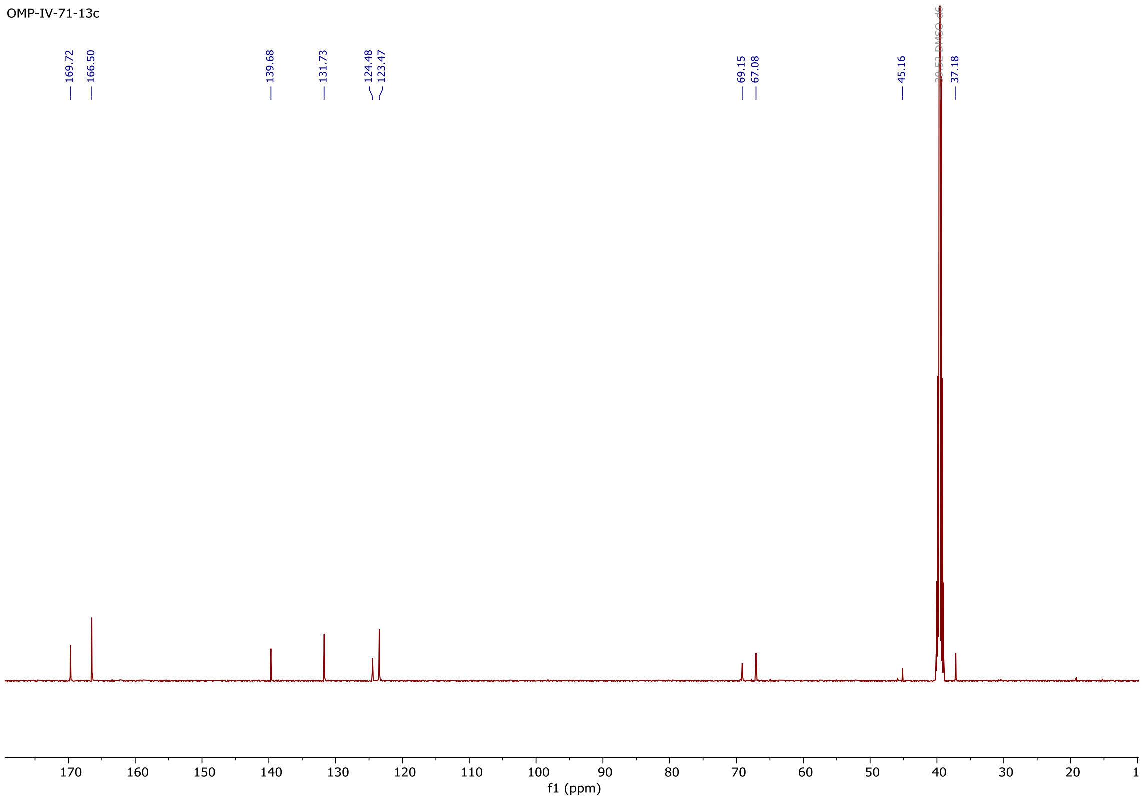

MS Spectrum

Compound Table

Compound Label	RT	Mass	Abund	Formula	Tgt Mass	Diff (ppm)
Cpd 1: C49 H48 N4 O24	0.344	1076.26573	389	C49 H48 N4 O24	1076.26585	-0.11

Compound Label	RT	Algorithm	Mass
Cpd 1: C49 H48 N4 O24	0.344	Find By Formula	1076.26573

MS Zoomed Spectrum

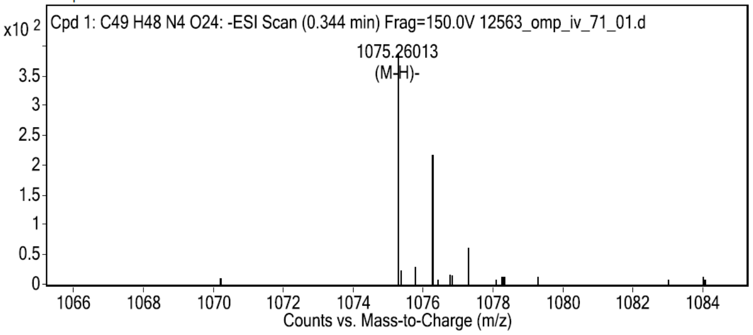

Intermediate 37

^{1}H NMR

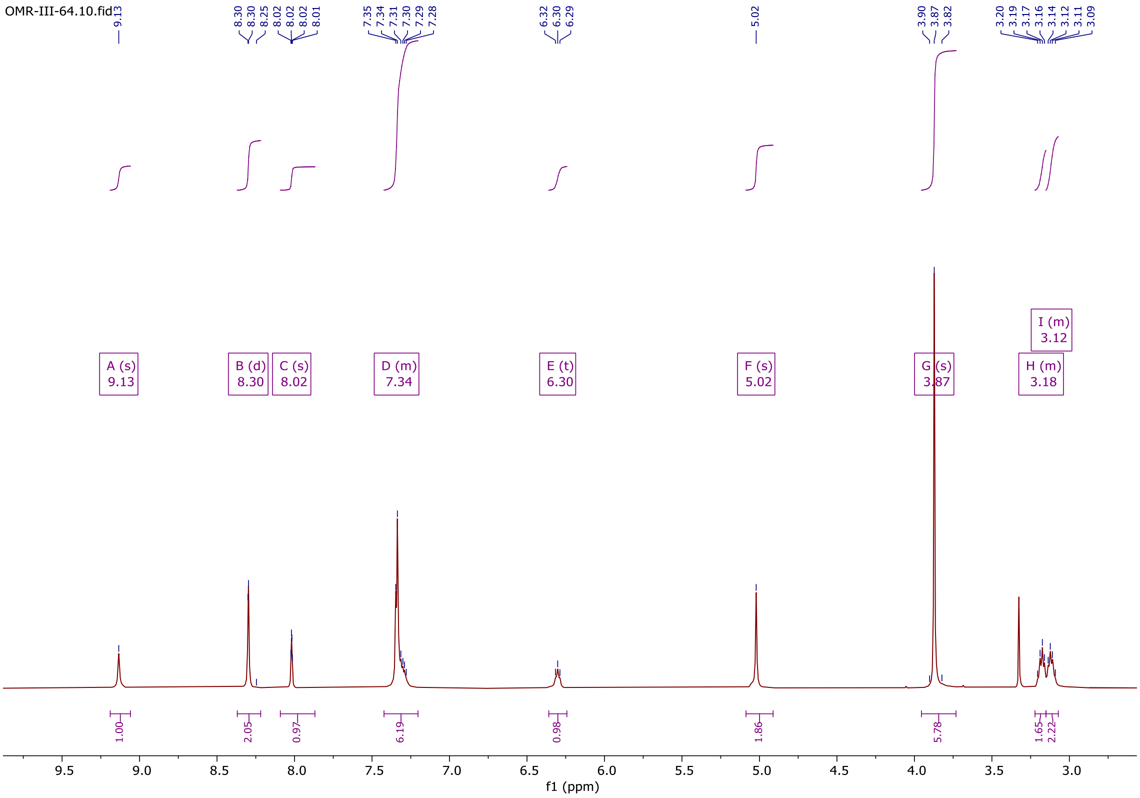

Intermediate 38

^{1}H NMR

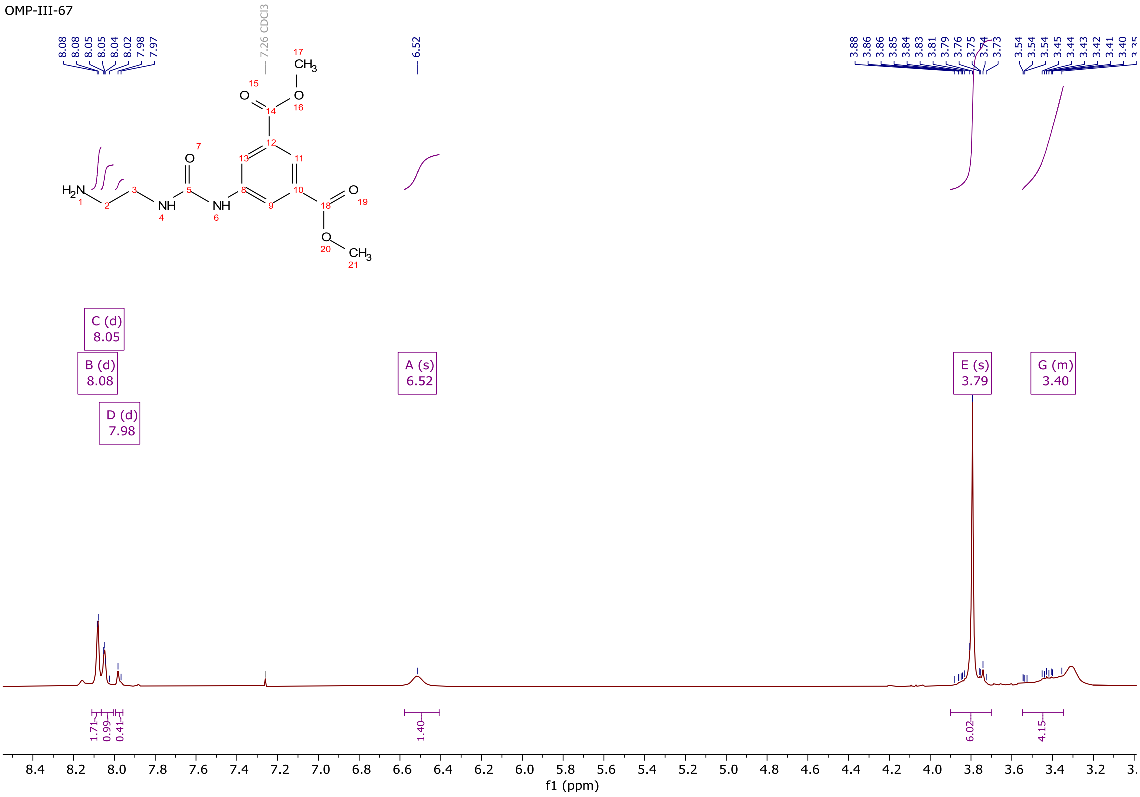

Tetramer 39

^{1}H NMR

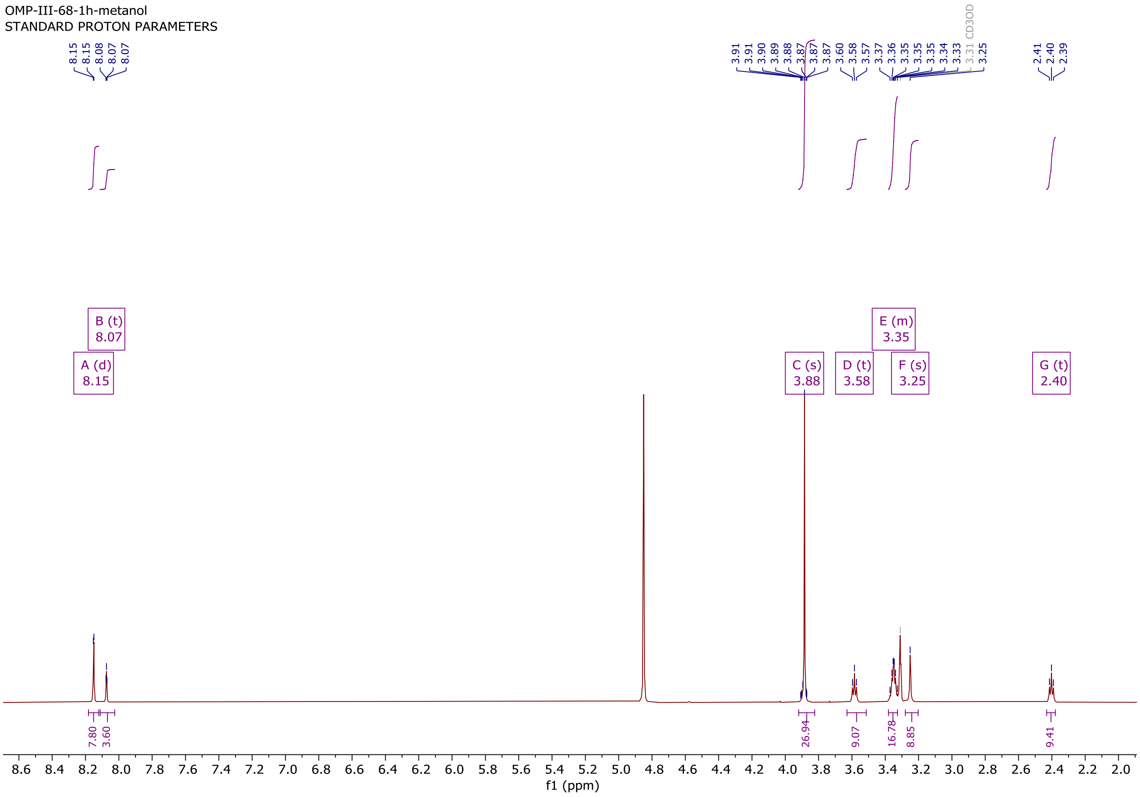

Tetramer 40

^{1}H NMR

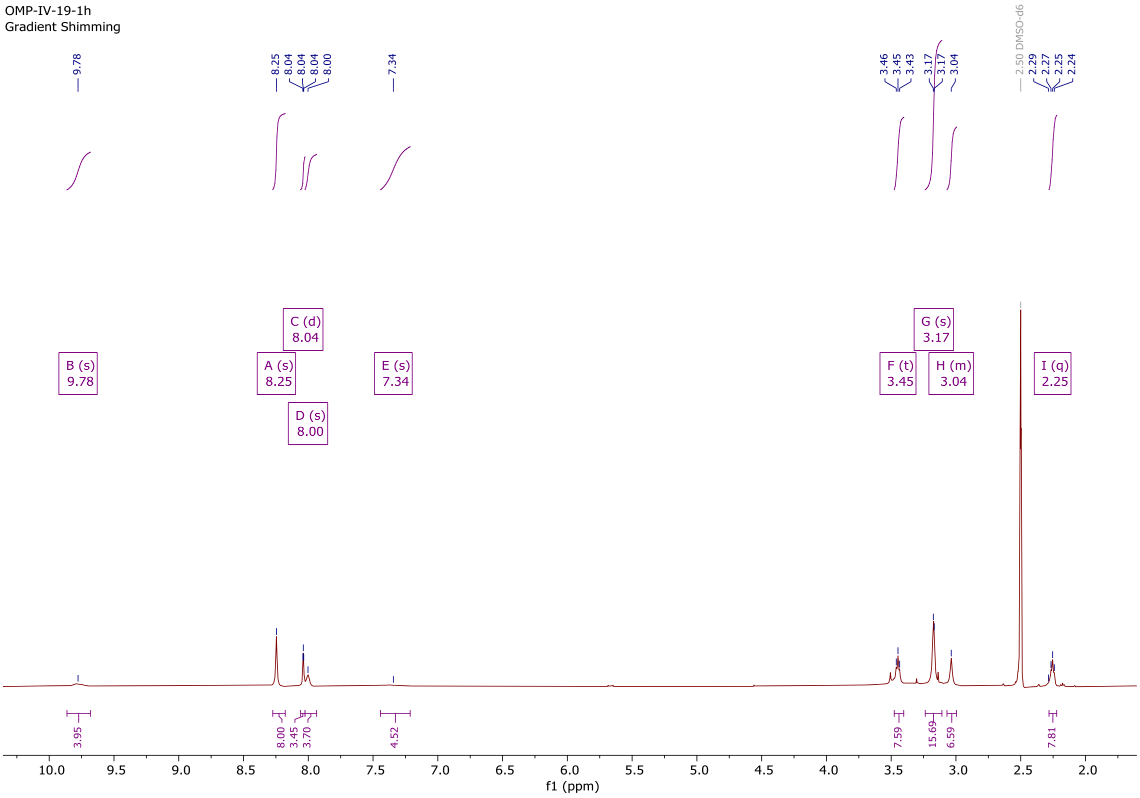

^{13}C NMR

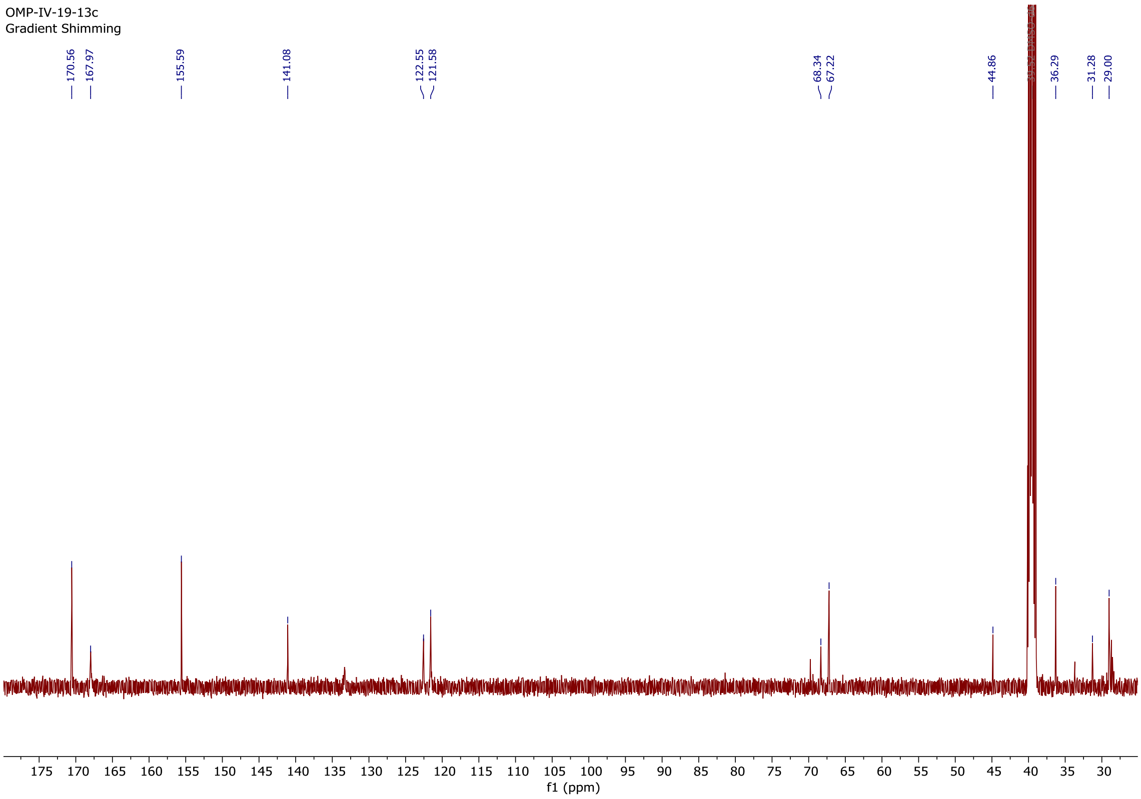

MS Spectrum

Compound Table

Compound Label	RT	Mass	Abund	Formula	Tgt Mass	Diff (ppm)
Cpd 1: C61 H72 N12 O28	0.203	1420.45595	2528	C61 H72 N12 O28	1420.4579	-1.37

Compound Label	RT	Algorithm	Mass
Cpd 1: C61 H72 N12 O28	0.203	Find By Formula	1420.45595

MS Zoomed Spectrum

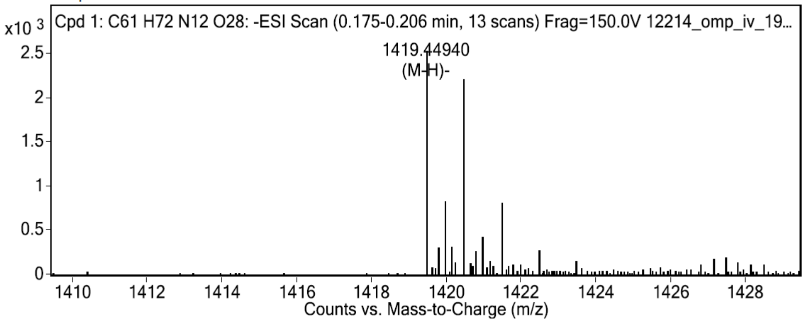

HPLC chromatogram

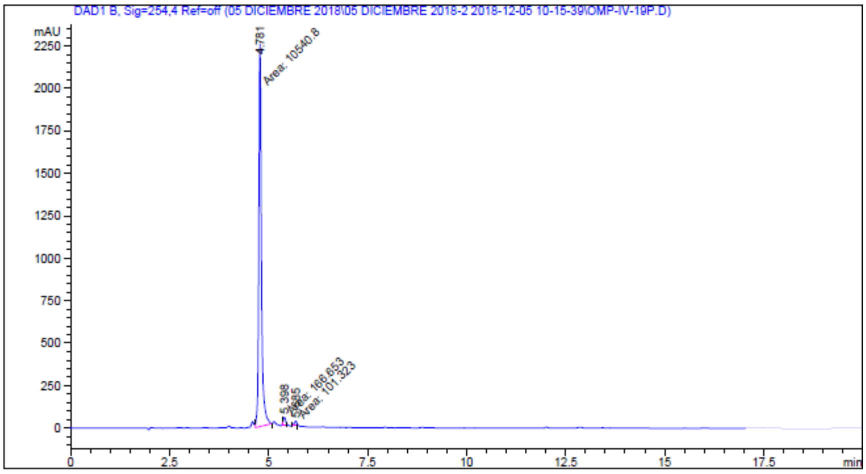


```
=====================================================================
                         Area Percent Report
=====================================================================

Sorted By             :      Signal
Multiplier:                    :      1.0000
Dilution:                      :      1.0000
Use Multiplier & Dilution Factor with ISTDs


Signal 1: DAD1 B, Sig=254,4 Ref=off

Peak RetTime Type  Width     Area      Height     Area
  #   [min]        [min]   [mAU*s]     [mAU]       %
----|-------|----|-------|----------|----------|--------|
   1   4.781 MM T  0.0777 1.05408e4 2259.69556  97.5208
   2   5.398 MM T  0.0589  166.65269   47.13331   1.5418
   3   5.685 MM T  0.0691  101.32336   24.45613   0.9374

Totals :                  1.08088e4 2331.28499
```

Figure S1

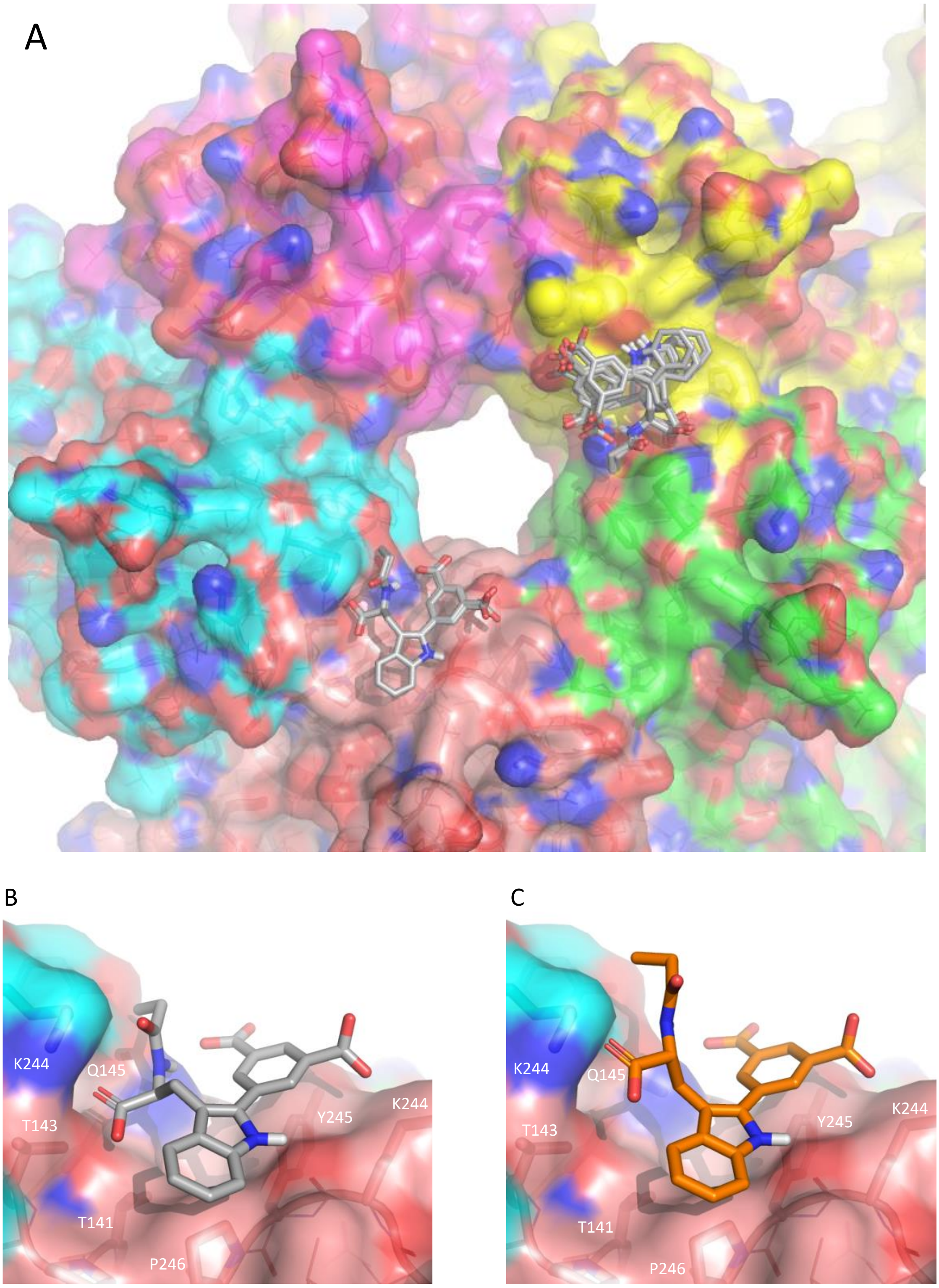
A
B
K244
Q145
T143
Y245
K244
T141
P246
C
K244
Q145
T143
Y245
K244
T141
P246

Figure S2

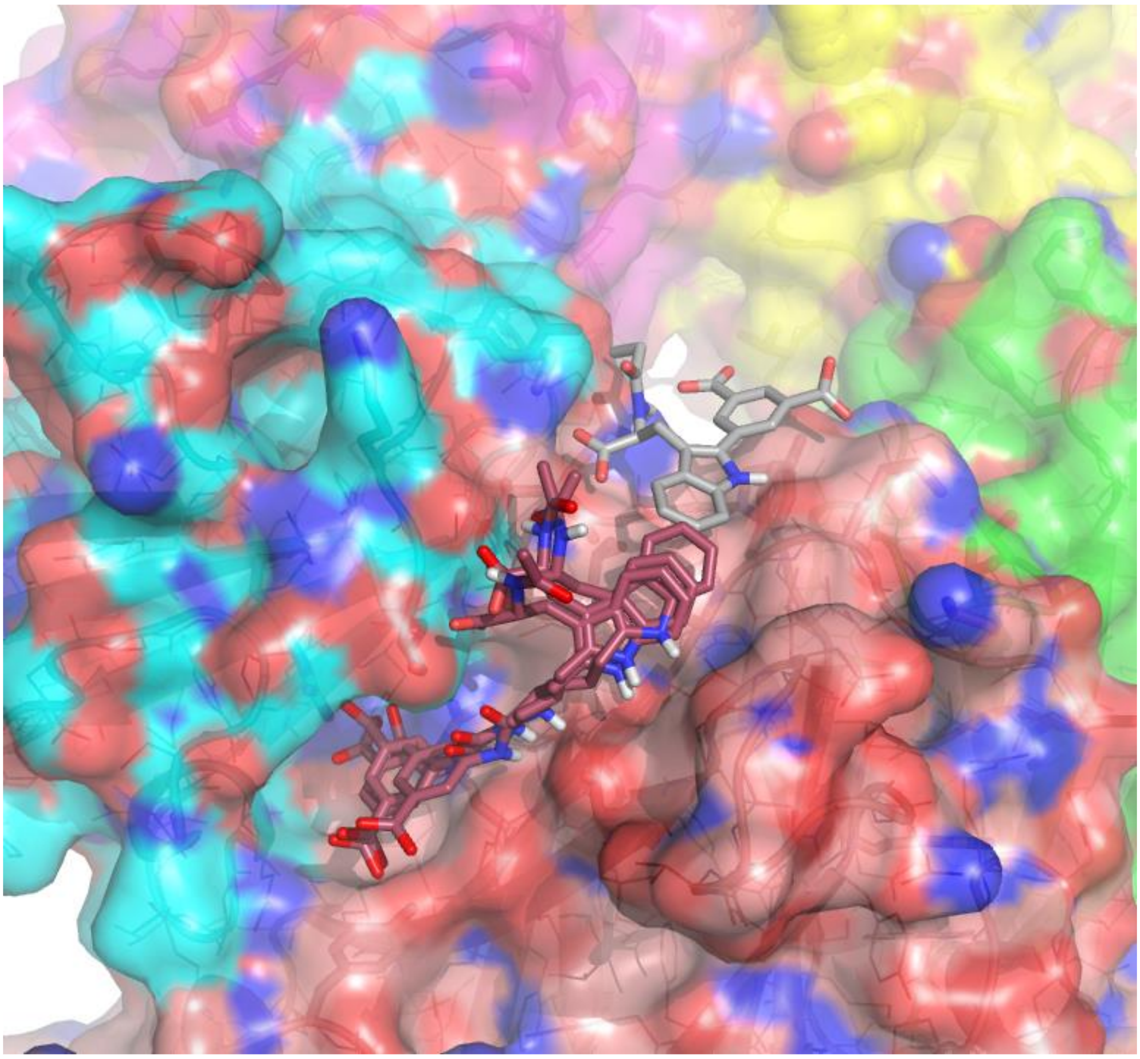

Figure S3

(**A**)

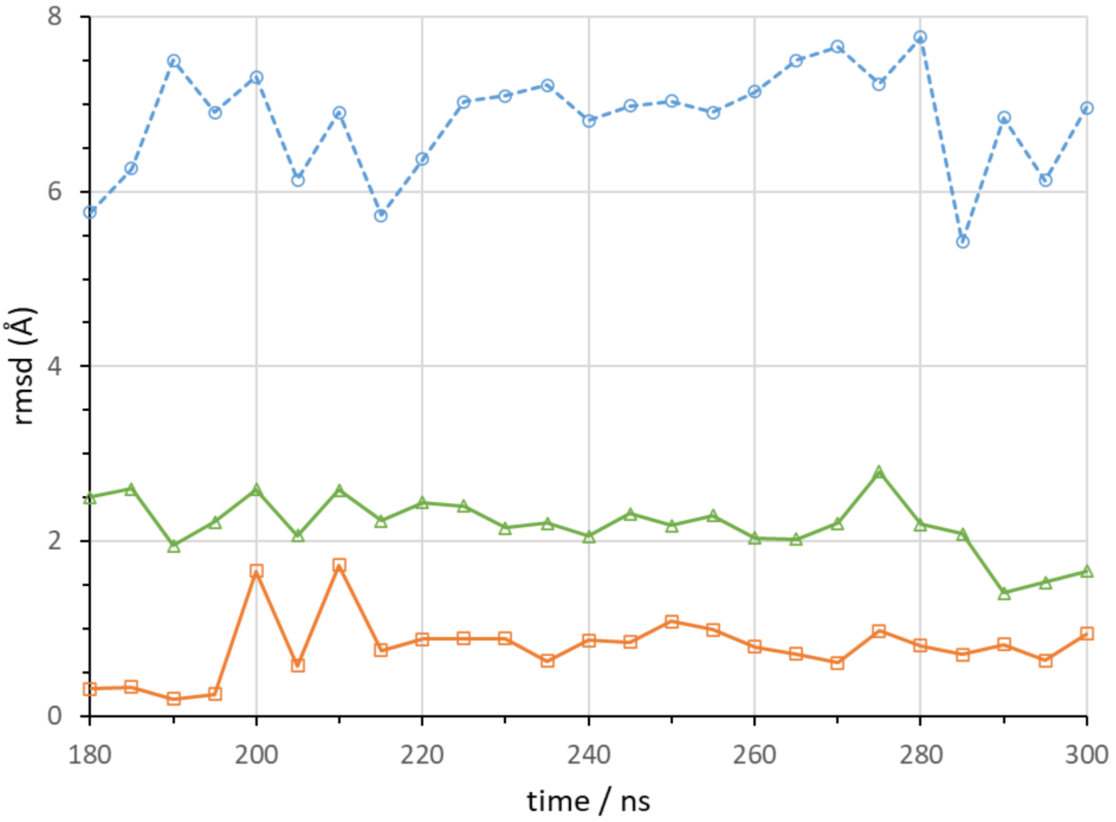

8
6
4
2
0
rmsd (Å)
180
200
220
240
260
280
300
time / ns

(**B**)

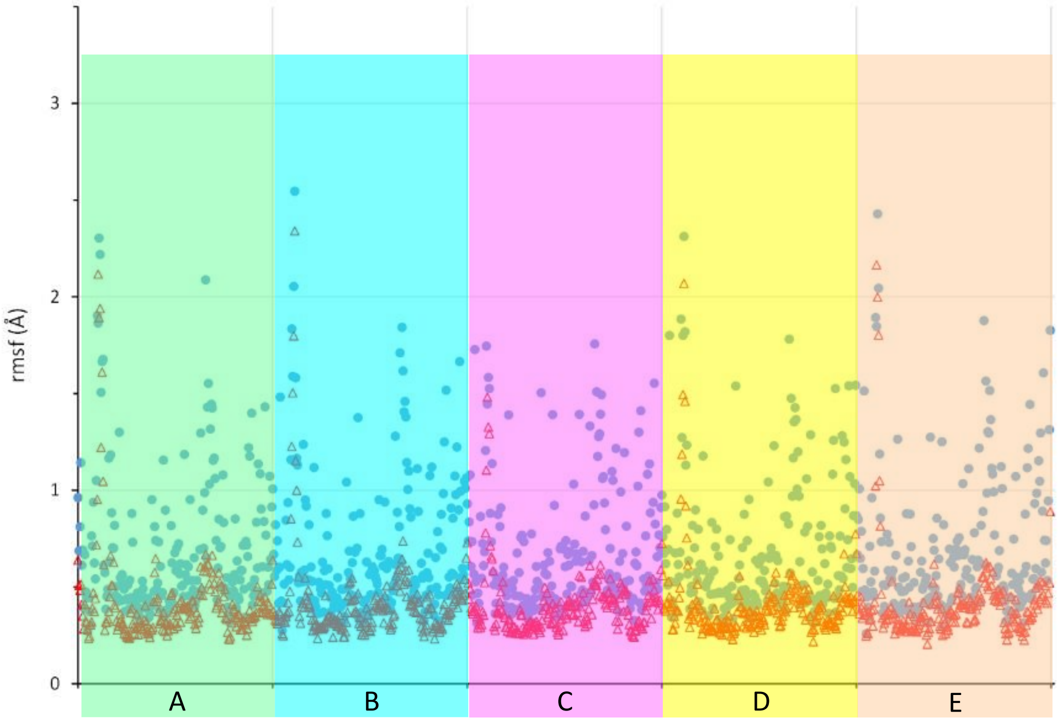

3
2
1
0
rmsf (Å)
A
B
C
D
E

Table S1. Predicted pharmacokinetic and toxicity properties

Property (Model name)	23 (AL-534) MW: 2226.203 Da	AL-471 MW: 1825.69 Da	Pirodavir MW: 369.465 Da
**Water solubility (log mol/L)**	-2.892	-2.892	-4.43
**Caco2 permeability (logPapp in 10^{-6} cm/s)**	-3.218	-2.32	1.46
**Intestinal absorption-human (% Absorbed)**	0	0	97.679
**Skin permeability (log Kp)**	-2.735	-2.735	-2.758
**Fraction unbound-human (Fu)**	0.381	0.381	0.049
**Blood–brain barrier permeability (log BB)**	-9.917	-7.212	0.23
**CNS Permeability (log PS)**	-8.257	-8.153	-2.463
**Total clearance (log mL/min/kg)**	-4.108	-3.41	0.549
**Maximum recommended tolerated dose (MRTD)-human (log mg/kg/day)**	0.438	0.438	0.679
**Oral rat accute toxicity (LD_{50}) (mol/kg)**	2.482	2.482	2.569
**Oral rat chronic toxicity-lowest observed adverse effect (LOAEL) (log mg/kg.bw/day)**	13.635	8.063	1.241
***T. Pyriformis* toxicity (log μg/L)**	0.285	0.285	0.538
**Flathead minnow toxicity (LC50) (log mM)**	3.313	-6.511	-0.293

The web server (https://biosig.lab.uq.edu.au/pkcsm/prediction) was used (Pires, D. E., Blundell, T. L., & Ascher, D. B. (2015). pkCSM: Predicting Small-Molecule Pharmacokinetic and Toxicity Properties Using Graph-Based Signatures. *J Med Chem, 58* 4066-4072. https://doi.org/10.1021/acs.jmedchem.5b00104).
